# The Effectiveness of Non‐Pharmacological Interventions on Preoperative and Postoperative Anxiety Among Patients Undergoing Abdominal Surgery: A Systematic Review and Meta‐Analysis

**DOI:** 10.1111/wvn.70099

**Published:** 2026-02-18

**Authors:** Wai Lin William Tse, Po Yan Sin, Kai Chow Choi, Ho Yu Cheng

**Affiliations:** ^1^ Department of Surgery North District Hospital Sheung Shui Hong Kong; ^2^ The Nethersole School of Nursing The Chinese University of Hong Kong Shatin Hong Kong

**Keywords:** abdominal surgery, anxiety, non‐pharmacological, pain, preoperative counseling, psychological intervention, relaxation exercise

## Abstract

**Background:**

Patients undergoing abdominal surgeries have a chance to experience surgical‐related anxiety. But the most effective non‐pharmacological interventions in managing this anxiety have not yet been identified.

**Aim(s):**

To examine the effectiveness of different types of non‐pharmacological interventions, and identify the effective components on pre‐ and postoperative anxiety management among patients undergoing abdominal surgeries.

**Methods:**

A systematic search of randomized control trials (RCTs) examined the effects of non‐pharmacological interventions on preoperative and/or postoperative anxiety (Primary outcomes) among patients undergoing abdominal surgery was conducted across MEDLINE, Ovid Nursing, AMED, PsycINFO, CINAHL, EMBASE, Cochrane Library, HyRead, and WANFANG DATA from 1987 to March 1, 2024. Secondary outcomes including postoperative pain, postoperative analgesics consumption, resumption of postoperative bowel movements, and length of hospital stay were also examined. Cochrane Risk of Bias Tool (version 2.0) was used for quality assessment. Meta‐analysis was performed to synthesize the findings. Narrative summaries were provided for the studies that could not be included in the meta‐analysis.

**Results:**

This review included 35 RCTs. The interventions of included studies were categorized as prehabilitation, sensory stimulation, preoperative counseling, information provision, and psychological interventions. Meta‐analysis revealed that preoperative counseling was beneficial in managing preoperative anxiety (SMD = −1.36; 95% CI = −1.96, −0.76), postoperative anxiety (SMD = −1.30; 95% CI = −1.62, −0.98), and postoperative pain (SMD = −0.84; 95% CI = −1.21, −0.47). Meanwhile, psychological interventions adopting relaxation exercises had potential effects in reducing postoperative opioid consumption and shortening time to postoperative bowel movement.

**Linking Evidence to Action:**

Adopting preoperative counseling is suggested for the management of pre‐ and postoperative anxiety and postoperative pain among patients undergoing elective abdominal surgeries. A one‐off lasting for 20–45 min preoperative counseling including individualized information about the coming surgery and perioperative process, and a discussion addressing patients' concerns is recommended. Future research is needed to explore the effects of relaxation exercise on important patients' outcomes such as postoperative analgesics consumption and time to resume bowel movement among patients undergoing abdominal surgery.

**Trial Registration:**

PROSPERO registration number: CRD42023359484

## 1 Introduction

Abdominal surgeries comprise a significant portion of elective and emergency surgery caseloads worldwide. Approximately 10 million abdominal surgeries were performed in the United States over a 5‐year period, while over 30,000 emergency laparotomies were conducted annually in the United Kingdom (Pirie et al. 2022). Also, in Asia, abdominal surgeries contribute to the largest proportion of endoscopic surgeries in Japan (Shiroshita et al. 2022), and over 80% of emergency surgeries among major and ultra‐major procedures in Hong Kong (Hospital Authority Quality and Safety Division 2021). However, the prevalence of anxiety among patients undergoing abdominal surgeries reached nearly 60% (Kassahun et al. 2022; Harms et al. 2023), which is higher than the global average prevalence rate of 50% among patients undergoing all types of surgeries (Abate et al. 2020).

Anxiety is a discomfort and tense feeling that usually comes from unknown or general causes (Abate et al. 2020). Patients undergoing abdominal surgeries who experience preoperative anxiety have been associated with several adverse outcomes during hospitalization, including increased postoperative pain (Liu et al. 2023), greater consumption of postoperative analgesics (Pekcan et al. 2023), increased risks for developing postoperative ileus (Banyong et al. 2022) and postoperative anxiety (Gümüs 2021), and prolonged hospital stays (Schlosser et al. 2019); thus, further impacting the quality of recovery (Gümüs 2021). Therefore, identifying effective means in managing pre‐ and postoperative anxiety among patients undergoing abdominal surgeries is urged.

Previous review on the management of anxiety among patients undergoing abdominal surgeries was mainly focused on a specific type of non‐pharmacological interventions, such as psychological interventions, without summarizing the effective components of the identified intervention (Villa et al. 2020). With the growing evidence on various non‐pharmacological interventions, such as aromatherapy (Beyliklioğlu and Arslan 2019) and massage (Ren et al. 2021), in managing pre‐ and/or postoperative anxiety, this systematic review aims to synthesize the best available evidence on the effectiveness of non‐pharmacological interventions in managing pre‐ and postoperative anxiety among patients undergoing abdominal surgeries.

## 2 The Review, Aims, and Methods

This review was registered in the PROSPERO (No. CRD42023359484) and was reported in accordance with the Preferred Reporting Items for Systematic Reviews and Meta‐Analyses (PRISMA) guidelines (Page et al. 2021). Two reviewers (WLT and PYS) conducted the data selection, data extraction, risk of bias assessment, and level of certainty assessment (refer to Appendix S2) independently. A third reviewer (HYC) was consulted to resolve any disagreements.

### 2.1 Aims

This review aims to (1) examine the effectiveness of different types of non‐pharmacological interventions on preoperative and/or postoperative anxiety (primary outcomes), postoperative pain, postoperative analgesics consumption, time to resume bowel movement, and length of hospital stays, and (2) identify the effective components of non‐pharmacological interventions for managing pre‐ and postoperative anxiety among patients undergoing abdominal surgeries.

### 2.2 Design and Search Methods

Published articles in English or Chinese were searched in MEDLINE, AMED, Ovid Nursing, CINAHL, EMBASE, Cochrane Library, PsycInfo, HyRead, and WANFANG DATA from 1987 to 1st March 2024, as the first article related to laparoscopic surgery was published in 1987 (Alkatout et al. 2021). The English keywords for the search (Table S1) were developed through a preliminary search and then translated into Chinese equivalents for the Chinese database search. A manual search was also conducted on the reference lists of the included studies and related reviews to identify additional relevant studies. Table S2 shows the search strategy on each database. All identified papers were managed using Covidence, following predefined criteria.

### 2.3 Inclusion Criteria

(1) Population: Adult patients (age > = 18) undergoing general abdominal surgeries, excluding urological, gynecological, and vascular surgeries; (2) Intervention: non‐pharmacological interventions aim at reducing preoperative anxiety (from the time of surgery acknowledgment to immediately before the procedure) and/or postoperative anxiety (from immediately after surgery completion to hospital discharge); (3) Control: usual standard perioperative care, or placebo control; (4) Outcomes: patient‐reported preoperative and/or postoperative anxiety; and (5) Study design: randomized controlled trials (RCTs).

### 2.4 Exclusion Criteria

Studies did not conduct subgroup analysis for patients undergoing abdominal surgeries if they also included patients undergoing other types of surgeries.

### 2.5 Risk of Bias Assessment

The risk of bias of all included trials was assessed using the Cochrane Risk of Bias Assessment tool (version 2). It contains five domains including randomization process, deviations from intended interventions, missing outcome data, measurement of the outcomes, and selection of reporting bias (Higgins et al. 2022). Each domain was assessed and rated as low risk, some concerns, or high risk, and an overall risk of bias for each study was determined.

### 2.6 Data Extraction

The details of the included studies were extracted using a self‐developed data extraction form, including authors, year, categories, study locations, types of surgeries, sample sizes, intervention details, and study results (closest to the time of surgery). The authors of the included study were contacted if further information was needed.

### 2.7 Data Synthesis

The meta‐analysis was conducted using Review Manager 5.4 for each outcome, if at least two similar studies were available. For the continuous data, standard mean difference (SMD) with 95% confidence intervals (CI) were calculated. The interpretation of SMD followed Cohen (1992), where 0.2, 0.5, and 0.8 indicate small, moderate, and large effect sizes, respectively. Given the observed heterogeneity in the interventions and anxiety measurement tools across the included studies, a random‐effects model was employed for the meta‐analyses (Higgins et al. 2022). The *I* ^2^ statistic was adopted to quantify heterogeneity of effect estimates across the included studies in meta‐analysis. An *I* ^2^ > 50% indicates considerable heterogeneity (Higgins et al. 2022). Narrative summaries were provided for those studies that provided inadequate information for a meta‐analysis. Publication bias of the meta‐analysis on primary outcomes was assessed through funnel plot visualization with Egger's regression tests.

## 3 Results

The PRISMA flow diagram showing the study selection process was illustrated in Figure S1. A total of 766 articles were initially yielded after searching nine databases; 12 additional studies that met the inclusion and exclusion criteria were identified. After removing duplicates and screening the titles and abstracts, the full texts of 239 studies were retrieved for further assessment of their eligibility. Finally, 35 studies were included.

### 3.1 Risk of Bias

Thirty‐two studies were rated as having some concerns, while three studies were rated as low risk of bias (Figure S2). Twenty studies provided insufficient information on allocation concealment and thus were rated as some concern in the randomization process. Twenty‐one studies were rated some concerns regarding deviations from the planned intervention due to either lack of adequate information to assess deviations from the assigned intervention, or failure to adhere to the intention‐to‐treat principle or use appropriate analyses. Regarding the missing outcome data, all studies were rated a low risk of bias. However, most studies were rated as some concern in outcome measurement based on their study designs, as all studies measured anxiety using patient‐reported questionnaires, but only six studies minimized the risk of bias by using placebo control. Sixteen studies were rated as some concerns about the selection of reporting bias due to the absence of information for trial registration.

### 3.2 Characteristics of Included Studies

All the studies included in this review were conducted across multiple countries or regions, including Turkey (*n* = 10; Akelma et al. 2020; Aktaş and İlgin 2023; Bulut and Karabulut 2023; Demir and Saritas 2020; Goktas et al. 2022; Menevşe and Yayla 2024; Ozhanli and Akyuz 2022; Soylu and Kartın 2021; Toğaç and Yılmaz 2021; Ugras et al. 2023), Iran (*n* = 7; Abbasnia et al. 2023; Amini et al. 2019; Hasanpour‐Dehkordi et al. 2019; Pasyar et al. 2020; Sadati et al. 2013; Valiee et al. 2012; Yadegari et al. 2021), China (*n* = 4; Fan and Wang 2018; Liao and Zhu 2022; Lu and Zhang 2018; Yu and Wang 2018), Taiwan (*n* = 3; Lin and Wang 2005; Lu et al. 2022; Tsay et al. 2008), Brazil (*n* = 2; Felix et al. 2018; Garcia et al. 2018), Singapore (*n* = 2; Lim et al. 2011; Lim et al. 2019), Australia (*n* = 1; Tou et al. 2012), Canada (*n* = 1; Cassin et al. 2016), Germany (*n* = 1; Klaiber et al. 2018), Norway (*n* = 1; Gade et al. 2014), Spain (*n* = 1; Barberan‐Garcia et al. 2018), Sweden (*n* = 1; Nilsson et al. 2005), and the United States (*n* = 1; Tusek et al. 1997). The number of participants in each study varied from 24 to 244. All study participants were undergoing elective abdominal surgeries, including upper and lower gastrointestinal surgery (*n* = 10), cholecystectomy or hernia repair (*n* = 14), liver surgery (*n* = 3), others (*n =* 2), and unspecified abdominal surgeries (*n* = 6).

Patient‐reported anxiety was assessed using a variety of validated self‐report tools across the included studies: State–Trait Anxiety Inventory—State form (*n* = 19), Hospital Anxiety and Depression Scale—Anxiety subscale (*n* = 5), Visual Analog Scale (*n* = 3), Anxiety Specific to Surgery Questionnaire (*n* = 2), State–Trait Anxiety Inventory—full form (*n* = 2), Beck Anxiety Inventory (*n* = 1), Generalized Anxiety Disorder‐7 (*n* = 1), Numeric Rating Scale (*n* = 1), and Self‐Rating Anxiety Scale (*n* = 1). Preoperative anxiety was typically measured within 24 h before surgery (*n* = 18), 1–3 days prior to surgery (*n* = 1), 1–2 months prior to surgery (*n* = 2), or without a clearly specified time (*n* = 1). Postoperative anxiety was typically assessed within 24 h after surgery (*n* = 10), on postoperative day two (*n* = 1), day three (*n* = 2), day five (*n* = 2), day seven (*n* = 1), 1–3 days post‐surgery (*n* = 1), or without a clearly specified time (during postoperative admission to the intensive care unit; *n* = 1). Excluding baseline measurements, post‐intervention anxiety was assessed once in most studies (*n* = 26), twice in some (Fan and Wang 2018; Klaiber et al. 2018; Yu and Wang 2018; Toğaç and Yılmaz 2021), three times in others (Lim et al. 2011, 2019), four times in a few (Tou et al. 2012; Bulut and Karabulut 2023), and seven times in one (Tusek et al. 1997).

### 3.3 Characteristics of Study Interventions

The interventions of the included studies were categorized into five types of interventions based on their intervention components. For sensory stimulation, 13 included studies focused on sensory stimuli, which included tactile (*n* = 6), auditory (*n* = 2), olfactory (*n* = 2), gustatory (*n* = 1), and visual stimulation (*n* = 2). Preoperative counseling is an interactive process in which healthcare professionals engage with patients to provide individualized, surgery‐related information (e.g., procedure details, risks, benefits, and recovery expectations) while also addressing and alleviating specific surgery‐related concerns or anxieties before the operation (Handoll and Parker 2008); it emphasizes two‐way communication to tailor support to the patient's needs. Six studies were categorized as preoperative counseling and one included study (Lim et al. 2019) additionally conducted postoperative telephone follow‐ups. In contrast, information provision refers to a one‐way delivery of surgery‐related details (e.g., via educational materials, videos, or verbal explanations) without explicit opportunities for patients to discuss or seek clarification on their concerns, distinguishing it from counseling by lacking an interactive component focused on emotional or psychological support. Four studies were categorized as information provision in this review. As per psychological interventions which comprised any psychological strategies in managing anxiety, 11 studies were included, such as relaxation exercise (*n* = 8), preoperative discussion (*n* = 1), and cognitive behavioral intervention (*n* = 2). The remaining study was categorized as prehabilitation, a multimodal preoperative intervention aimed at optimizing patients' physical and functional status to reduce perioperative complications and enhance recovery; it typically includes supervised endurance exercise training to improve aerobic capacity, along with elements like nutritional guidance, smoking cessation, and encouragement of physical activity participation (Carli and Zavorsky 2005), prioritizing proactive physical preparation over informational or emotional support. Table S3 provides a summary of the characteristics of the included studies.

### 3.4 Effectiveness of the Interventions

#### 3.4.1 Preoperative Anxiety

Twenty‐two included studies examined preoperative anxiety, of which 18 examined preoperative anxiety within 24 h prior to the surgery, and 16 of these studies were included in the meta‐analysis (Figure S3a). Two studies were excluded because of inadequate information (Tusek et al. 1997; Sadati et al. 2013). The overall effect of the non‐pharmacological interventions on preoperative anxiety management was large (SMD = −0.80; 95% CI = −1.10, −0.51; *I* ^2^ = 84%). In the meta‐analysis, the most effective intervention was preoperative counseling with a large combined effect in two studies (SMD = −1.36; 95% CI = −1.96, −0.76; *I* ^2^ = 75%). For the studies that could not be included in the meta‐analysis, psychological interventions (Tusek et al. 1997), and preoperative counseling (Sadati et al. 2013) demonstrated a statistically significant effect (*p* < 0.05).

Regarding the remaining four studies, preoperative anxiety was examined either 1–3 days prior to surgery (Lin and Wang 2005), 1–2 months before surgery (Gade et al. 2014; Cassin et al. 2016), or without a clearly specified timing (Barberan‐Garcia et al. 2018). However, inadequate information precluded the conduct of a meta‐analysis for these groups. Preoperative counseling in Lin and Wang (2005) study demonstrated a statistically significant moderate effect on reducing anxiety (Hedges' *g* = 0.65, *p* ≤ 0.001); so did psychological interventions in Gade et al. (2014) (Hedges' *g* = 0.62, *p* ≤ 0.001) and Cassin et al. (2016) (Cohens' *d* = 1.03, *p* < 0.001). In contrast, prehabilitation showed no statistically significant effect (*p* ≥ 0.05) (Barberan‐Garcia et al. 2018).

#### 3.4.2 Postoperative Anxiety

Eighteen included studies examined postoperative anxiety, of which 10 examined postoperative anxiety within postoperative 24 h and nine of these studies were included in the meta‐analysis (Figure S3b). One study was excluded because of inadequate information (Tusek et al. 1997). The overall effect of the non‐pharmacological interventions on postoperative anxiety management was large (SMD = −0.80; 95% CI = −1.11, −0.49; *I* ^2^ = 74%). In the meta‐analysis, the most effective intervention was preoperative counseling with a large combined effect in two studies (SMD = −1.30; 95% CI = −1.62, −0.98; *I* ^2^ = 0%). For the studies that could not be included in the meta‐analysis, psychological interventions showed a statistically significant effect (*p* < 0.05) (Tusek et al. 1997).

For the remaining eight studies, postoperative anxiety was measured either on postoperative day two (Lu et al. 2022), day three (Lim et al. 2019; Ozhanli and Akyuz 2022), day five (Tsay et al. 2008; Yu and Wang 2018), day seven (Klaiber et al. 2018), 1–3 days post‐surgery (Lim et al. 2011), or without a clearly specified timing (during postoperative ICU admission; Demir and Saritas 2020). This variability precluded the conduct of a meta‐analysis. Sensory stimulation in three studies–Tsay et al. (2008) (Hedges' *g* = 0.77, *p* = 0.023), Yu and Wang (2018) (Cohen's *d* = 1.83, *p* < 0.001), and Demir and Saritas (2020) (Cohen's *d* = 1.35, *p* < 0.001)–and psychological interventions in two studies–Lu et al. (2022) (Cohen's *d* = 0.81, *p* = 0.01) and Ozhanli and Akyuz (2022) (Hedges' *g* = 1.13, *p* < 0.001)–demonstrated statistically significant effects on reducing anxiety. In contrast, preoperative counseling focused on stoma education (Lim et al. 2019) and information provision (Lim et al. 2011; Klaiber et al. 2018) showed no statistically significant effects (*p* ≥ 0.05).

#### 3.4.3 Postoperative Pain

Seventeen included studies examined postoperative pain, of which 11 examined postoperative pain within postoperative 24 h and five studies were included in the meta‐analysis (Figure S3c). Six studies were excluded because of inadequate information (Tusek et al. 1997; Sadati et al. 2013; Hasanpour‐Dehkordi et al. 2019; Akelma et al. 2020; Soylu and Kartın 2021; Toğaç and Yılmaz 2021). The overall effect of the non‐pharmacological interventions on postoperative pain management was moderate (SMD = −0.73; 95% CI = −0.95, −0.51; *I* ^2^ = 13%). The most effective intervention was also preoperative counseling with a large combined effect in two studies (SMD = −0.84; 95% CI = −1.21, −0.47; *I* ^2^ = 0%). For those studies that could not be included in the meta‐analysis, only auditory stimulation (Akelma et al. 2020) showed no statistically significant effect. Three other interventions including preoperative counseling (Sadati et al. 2013; Toğaç and Yılmaz 2021) (*p* ≤ 0.001), tactile stimulation (Soylu and Kartın 2021) (*p* = 0.042), and psychological interventions (Tusek et al. 1997; Hasanpour‐Dehkordi et al. 2019) (*p* < 0.05) demonstrated statistically significant improvement on postoperative pain.

For the remaining six studies, postoperative pain was examined either on postoperative day two (Klaiber et al. 2018; Lu et al. 2022), day three (Tsay et al. 2008; Ozhanli and Akyuz 2022), day five (Yu and Wang 2018), or without a clearly specified timing (during postoperative ICU admission; Demir and Saritas 2020). This variability precluded the conduct of a meta‐analysis. Sensory stimulation applied in three studies–Tsay et al. (2008) (Hedges' *g* = 0.85, *p* < 0.001), Yu and Wang (2018) (Cohen's *d* = 0.63, *p* < 0.05), and Demir and Saritas (2020) (Cohen's *d* = 1.76, *p* = 0.000)–as well as psychological interventions employed in two studies–Lu et al. (2022) (Cohen's *d* = 0.52, *p* < 0.001) and Ozhanli and Akyuz (2022) (Hedges' *g* = 1.55, *p* = 0.000)–demonstrated a statistically significant effect. In contrast, information provision (Klaiber et al. 2018) showed no statistically significant effect (*p* ≥ 0.05).

#### 3.4.4 Postoperative Analgesic Consumption

Seven included studies examined postoperative analgesic consumption. However, a meta‐analysis could not be conducted due to inadequate information and different types of analgesics consumed, such as opioid (*n* = 5), non‐steroidal anti‐inflammatory drugs (NSAIDs, *n* = 2), acetaminophen (*n* = 1), and unspecified analgesics (*n* = 1). Six out of seven studies reported a statistically significant effect on reducing postoperative opioid, acetaminophen, or unspecified analgesic consumption (*p* < 0.05). These interventions included sensory stimulation (Nilsson et al. 2005; Tsay et al. 2008; Fan and Wang 2018; Liao and Zhu 2022) and psychological interventions adopting relaxation exercises (Tusek et al. 1997; Ozhanli and Akyuz 2022), in which only three studies provided effect size, including Nilsson et al. (2005) showing a moderate effect size (Cohen's *d* = −0.74), Tsay et al. (2008) showing a moderate effect size (Hedges' *g* = −0.64), and Ozhanli and Akyuz (2022) showing a large effect size (Hedges' *g* = −0.81). However, psychological interventions adopting relaxation exercises (Lu et al. 2022; Ozhanli and Akyuz 2022) showed no statistically significant effect on NSAIDs' consumption (*p* ≥ 0.05).

#### 3.4.5 Resumption of Postoperative Bowel Movement

Four included studies examined the duration for postoperative bowel returns. Only psychological interventions adopting relaxation exercise reported a statistically significant effect (*p* < 0.05) (Tusek et al. 1997). Meanwhile, tactile stimulation (Fan and Wang 2018; Soylu and Kartın 2021) and preoperative counseling (Sadati et al. 2013) showed no statistically significant effect (*p* ≥ 0.05).

#### 3.4.6 Length of Hospital Stay

Eight included studies examined the length of hospital stay, and none of these studies provided adequate information for conducting a meta‐analysis (Tusek et al. 1997; Sadati et al. 2013; Barberan‐Garcia et al. 2018; Fan and Wang 2018; Klaiber et al. 2018; Yu and Wang 2018; Lim et al. 2019; Liao and Zhu 2022). All five types of interventions showed no statistically significant effects (*p* ≥ 0.05).

### 3.5 Publication Bias

Publication bias for the meta‐analysis of primary outcomes was examined. The funnel plots of each meta‐analysis (Figure S4) appeared symmetrical and the *p*‐value from Egger's regression test for pre‐ and postoperative anxiety were 0.705 and 0.754, respectively, indicating that no publication bias was observed.

## 4 Discussion

The findings of this review pooled from 35 RCTs showed that non‐pharmacological interventions demonstrated large combined effects in managing both pre‐ and postoperative anxiety among patients undergoing abdominal surgeries. Among the five types of interventions, preoperative counseling showed the largest effect on reducing preoperative anxiety, postoperative anxiety, and postoperative pain within postoperative 24 h. Regarding postoperative opioid consumption and resumption of postoperative bowel movement, psychological interventions adopting relaxation exercises showed potential effects. However, none of the identified interventions showed beneficial effects on length of hospital stay.

Comparing the findings of the current review and a previous review by Villa et al. (2020), they suggested that psychological interventions were generally beneficial for managing preoperative anxiety and postoperative pain in patients undergoing abdominal surgeries. In addition, the findings of this review concluded that preoperative counseling is effective in managing pre‐ and postoperative anxiety and postoperative pain among patients undergoing abdominal surgeries along with their effective components. Besides, the findings of this review also highlighted how they were effective, as indicated by the provided effect size. Furthermore, this review included postoperative analgesic consumption, resumption of postoperative bowel movement, and length of hospital stay, which are significant patients' outcomes after abdominal surgery as the secondary outcomes which were not included in Villa et al. (2020) review. Therefore, the findings of this review also supplemented that relaxation exercise which targeted preoperative and/or postoperative anxiety management has potential effects on reducing postoperative opioid consumption and shortening time to resume postoperative bowel movements.

Based on the findings of this review, a one‐off preoperative counseling lasting 20–45 min after admission is recommended. The common contents should include individualized information about the upcoming surgery and the perioperative process, such as pain management and perioperative care, with the supplement of audiovisual or written materials. At last, the concerns from patients should be addressed as these were the most important element when compared to information provision alone. On the other hand, relaxation exercises incorporating approaches such as mental focus via imagery or progressive muscle relaxation, performed 1–2 times for 15–20 min daily, both before and after the surgery, were found to have potential effects on reducing opioid consumption and shortening the time to resumption of bowel movement after surgery. Before the first implementation, nurses should explain its importance and benefits and assess the patients' readiness for the self‐implementation. More research is required to explore these effects among patients undergoing abdominal surgery.

Nurses were the first contact person during admission in a surgical ward. They had much contact time and developed close relationships with these patients during providing perioperative care (Majumdar et al. 2019). Therefore, nurses should be the most suitable resource person to deal with surgical‐related anxiety among patients undergoing abdominal surgeries. With the right time, dose, and format of the non‐pharmacological interventions, such as preoperative counseling and relaxation exercises, nurses can help these patients not only minimize their pre‐ and postoperative anxiety and postoperative pain but also potentially reduce their postoperative opioid consumption and promote the resumptions of their postoperative bowel movements. Therefore, incorporating preoperative counseling with relaxation exercises in the perioperative nursing care among patients undergoing abdominal surgeries can be considered.

### 4.1 Strengths, Limitation, and Implications

This review had strengths that contributed to its value. First, this review not only incorporated pre‐ and postoperative anxiety management as the primary outcomes but also intended to include some objective patient outcomes during hospitalization as the secondary outcomes, such as postoperative analgesic consumption, time to resume postoperative bowel movement, and length of hospital stay, which were mostly strongly associated with surgical‐related anxiety and were critical patients' outcomes in abdominal surgeries. Secondly, this review analyzed the effects on anxiety management based on the measuring time of anxiety, either before (from the time of surgery acknowledgment to immediately before the procedure) or after the surgery (from immediately after surgery completion to hospital discharge). As a result, the types of non‐pharmacological interventions that effectively manage pre‐ and postoperative anxiety could be identified, respectively.

For the limitations, this review provided limited generalizability among patients undergoing emergency abdominal surgeries. Although we included both emergency and elective abdominal surgeries, only elective abdominal surgeries were finally identified in this review. This showed the management of anxiety in patients undergoing emergency abdominal surgeries may not be sufficiently focused. However, due to the emergency nature, the patients undergoing emergency surgeries were more likely to have preoperative anxiety than those undergoing elective ones (Woldegerima, et al. 2022). Therefore, future studies should address this knowledge gap. Besides, although we had tried to contact the authors for more information, several studies provided inadequate data for estimating standard deviations or standard errors, which impeded the completion of certain meta‐analyses. In addition, 32 of the included studies were rated as having some concerns in the risk of bias assessment. Therefore, more rigorous studies should be conducted that provide adequate information, such as details on randomization and allocation concealment, means and standard deviations, and incorporate placebo controls to minimize bias, thereby improving the quality of evidence.

The lack of standardized timing and frequency in anxiety assessments across the included studies posed a significant limitation, as it prevented the conduct of certain subgroup or time‐point‐specific meta‐analyses (e.g., comparisons across multiple perioperative days). This variability was addressed in part through the random‐effects model, but it highlights the need for greater consistency in future protocols. Accordingly, future research should prioritize examining intervention effects on surgical anxiety using a regular, predefined assessment schedule to enhance comparability and enable more robust syntheses of evidence. Lastly, limited studies examining the intervention's effect on preoperative and/or postoperative anxiety included important patient outcomes such as postoperative analgesics consumption, time for resuming postoperative bowel movement, and length of hospital stays, which made the conduct of certain meta‐analyses impossible. Future studies targeting preoperative and/or postoperative anxiety management are recommended to examine these important patients' outcomes in abdominal surgeries. The interpretation of the findings should be approached cautiously, considering these limitations.

### 4.2 Linking Evidence to Action

- To effectively manage pre‐ and postoperative anxiety and postoperative pain in patients undergoing abdominal surgeries, utilization of preoperative counseling is recommended. A one‐off preoperative counseling session including individualized information about the coming surgery and the perioperative process, and a discussion to address the patient concern, lasting for 20–45 min is recommended.
- Relaxation exercises incorporating mental focus via imagery or progressive muscle relaxation–performed 1–2 times daily for 15–20 min both before and after surgery–demonstrated potential effects on reducing postoperative opioid consumption and shortening the time to resumption of bowel movements; further studies are warranted to confirm and expand on these findings.
- Future studies targeting preoperative and/or postoperative anxiety management should also examine the effects of important patient outcomes such as length of hospital stay, postoperative analgesic consumption, and time for resuming postoperative bowel movement, and aim to manage the pre‐ and postoperative anxiety in patients undergoing emergency abdominal surgeries.

## 5 Conclusion

The findings of this review indicate that non‐pharmacological interventions are overall effective in managing pre‐ and postoperative anxiety and postoperative pain in patients undergoing abdominal surgeries. Among the five types of non‐pharmacological interventions examined, preoperative counseling showed a large effect size in managing preoperative anxiety (SMD = −1.36), postoperative anxiety (SMD = −1.30), and postoperative pain (SMD = −0.84). In addition, relaxation exercises potentially reduce postoperative opioid consumption and shorten the time for resuming postoperative bowel movement. Therefore, to manage pre‐ and postoperative anxiety and enhance the postoperative recovery among patients undergoing abdominal surgeries, incorporating preoperative counseling with relaxation exercises in the perioperative nursing care among patients undergoing abdominal surgeries can be considered.

## Funding

The authors have nothing to report.

## Ethics Statement

The systematic review is not subject to ethical review.

## Conflicts of Interest

The authors declare no conflicts of interest.

## Supporting information


**Appendix S1:** Supporting Information

**Appendix S2:** Supporting Information

**Figure S1:** PRISMA flow diagram showing the study selection process.
**Figure S2:** The results of risk of bias assessment of the included studies: (a) Risk of bias summary; and (b) Risk of bias graph.
**Figure S3:** Forest plot of the effectiveness of non‐pharmacological interventions among patients undergoing.
**Figure S4:** Funnel plots of the meta‐analyses of: (a) preoperative anxiety within.

**Table S1:** Identified keywords in PICOS form.
**Table S2:** Search strategy and initial results.
**Table S3:** Summary table of the characteristics of the included studies.

## Data Availability

The data that supports the findings of this study are available in Appendices [Link], [Link], Tables S1–S3, and Figures S1–S4 of this article.
