# Supplementary material for: The Effectiveness of Non‐Pharmacological Interventions on Preoperative and Postoperative Anxiety Among Patients Undergoing Abdominal Surgery: A Systematic Review and Meta‐Analysis
Source: Worldviews Evid Based Nurs. 2026 Feb 18;23(1):e70099. doi: 10.1111/wvn.70099 (PMC12917301; doi:10.1111/wvn.70099)
Supplement: Supplementary file 2 — Appendix S2: Supporting Information [file WVN-23-0-s001.docx]

**Supplementary information 1: Assessment on the quality of evidence**

The level of certainty for each outcome in the meta-analyses was assessed using the Grading of Recommendations, Assessment, Development, and Evaluation (GRADE) system (GRADE Working Group, 2004). It contains five domains including the risk of bias, inconsistency, indirectness, imprecision, and publication bias. The level of certainty ranged from high (++++), moderate (+++), low (++), and very low (+), with each factor potentially lowering the level of certainty by one degree if present of the above domains.

The meta-analyses conducted on the outcomes of preoperative anxiety and postoperative anxiety had a low quality of evidence, as most of the included studies were considered to have some concerns regarding the risk of bias, and there was considerable heterogeneity across the studies. On the other hand, the meta-analysis conducted on the outcomes of postoperative pain had a moderate quality of evidence due to some concerns regarding the risk of bias across the studies. Supplementary Information Table 1 presents the assessment of the level of certainty for the three outcomes.

| Supplementary Information Table 1. Summary table presenting the assessment on level of certainty | | | | | | | | | |
| --- | --- | --- | --- | --- | --- | --- | --- | --- | --- |
| Outcomes | No. of studies  (No. of participants) | Study  design | Risk of bias | Imprecision | Inconsistency | Indirectness | Publication bias | Other considerations | Certainty |
| Preoperative anxiety | 16 (1320) | RCT | Serious ^a^ | None ^b^ | Serious ^c^ | None ^e^ | None ^f^ | None | ++  Low |
| Postoperative anxiety | 9 (735) | RCT | Serious ^a^ | None ^b^ | Serious ^c^ | None ^e^ | None ^f^ | None | ++  Low |
| Postoperative pain | 5 (419) | RCT | Serious ^a^ | None ^b^ | Not serious ^d^ | None ^e^ | NA ^g^ | None | +++  Moderate |
| Abbreviations: RCT: Randomized Controlled Trial  Notes. ^a^ Most of the included studies were assessed as posing some concerns.  ^b^ Overall sample size >300.  ^c^ Considerable heterogeneity was noted.  ^d^ Low heterogeneity was noted  ^e^ No indirectness of population, intervention, comparison, or outcomes.  ^f^ p value of the Egger’s regression test ≥ 0.05.  ^g^ Publication bias was not assessed as postoperative pain was a secondary outcome. | | | | | | | | | |

**References**

GRADE Working Group. (2004). Grading quality of evidence and strength of

recommendations. *BMJ, 328*(7454), 1490. http://doi.org/10.1136/bmj.328.7454.1490
