# Supplementary material for: The Effectiveness of Non‐Pharmacological Interventions on Preoperative and Postoperative Anxiety Among Patients Undergoing Abdominal Surgery: A Systematic Review and Meta‐Analysis
Source: Worldviews Evid Based Nurs. 2026 Feb 18;23(1):e70099. doi: 10.1111/wvn.70099 (PMC12917301; doi:10.1111/wvn.70099)
Supplement: Supplementary file 3 — Figure S1: PRISMA flow diagram showing the study selection process. Figure S2: The results of risk of bias assessment of the included studies: (a) Risk of bias summary; and (b) Risk of bias graph. Figure S3: Forest plot of the effectiveness of non‐pharmacological interventions among patients undergoing. Figure S4: Funnel plots of the meta‐analyses of: (a) preoperative anxiety within. [file WVN-23-0-s004.pdf]

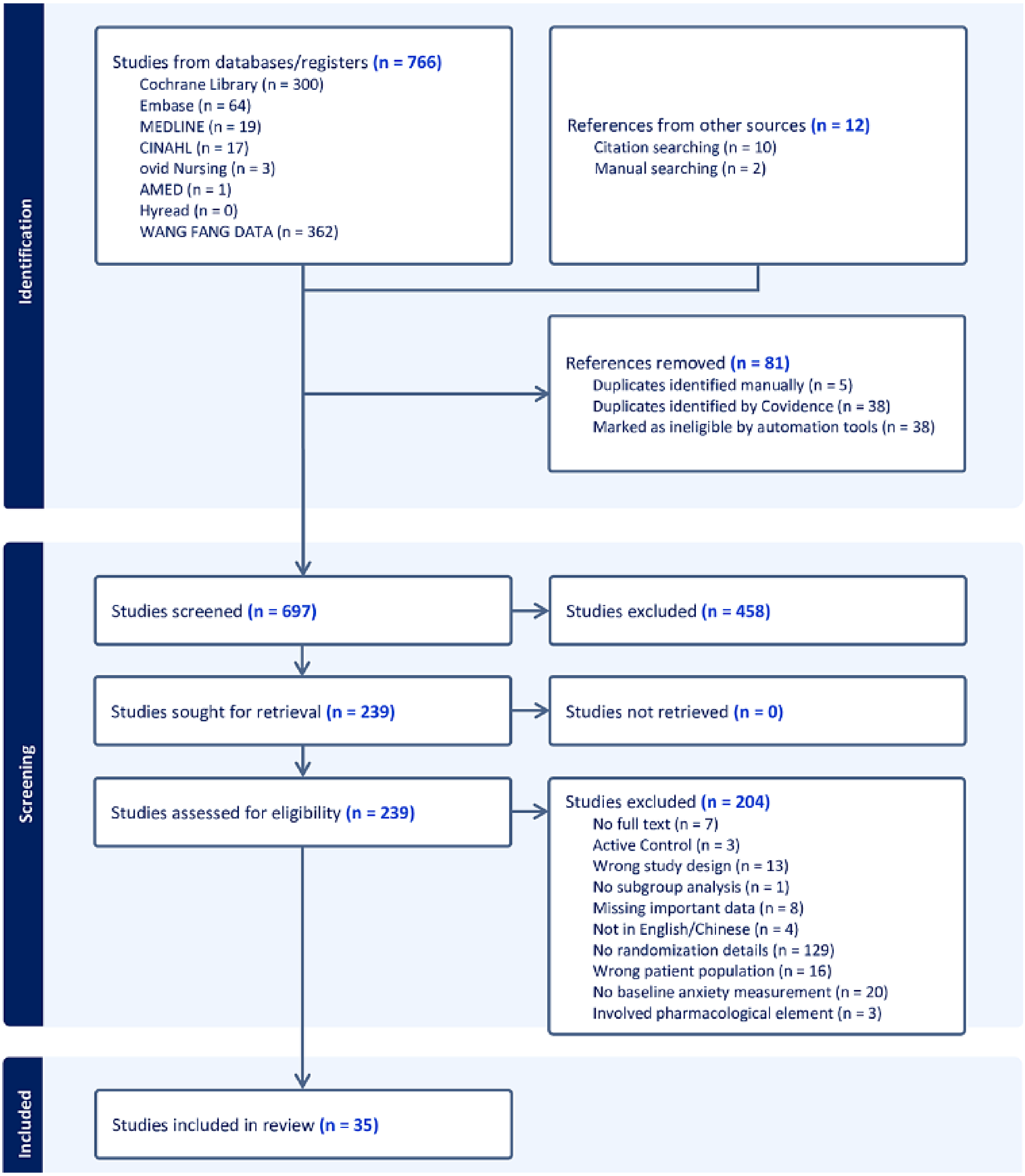

Figure S1. PRISMA flow diagram showing the study selection process.

(a) Risk of Bias Summary

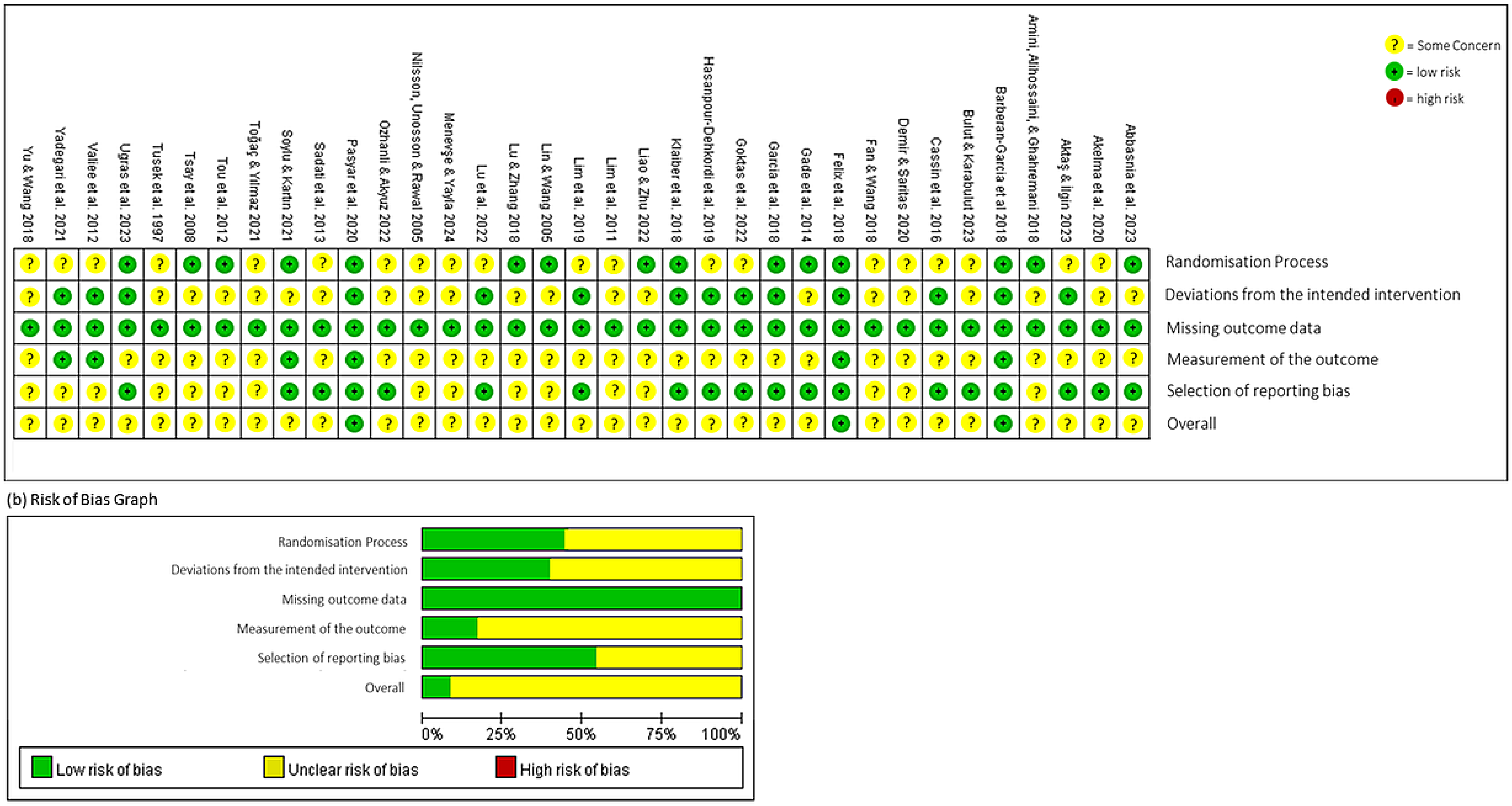

Figure S2: The results of risk of bias assessment of the included studies: (a) Risk of bias summary; and (b) Risk of bias graph.

(a) Preoperative Anxiety Within Preoperative 24 Hours

Study or Subgroup	Experimental Mean	Experimental SD	Experimental Total	Control Mean	Control SD	Control Total	Weight	Std. Mean Difference IV, Random, 95% CI	Year
**1.1.1 Sensory Stimulation**									
Valiee et al. 2012	4	1.27	35	5.07	1.17	35	6.3%	-0.87 [-1.36, -0.38]	2012
Fan & Wang 2018	46.84	2.95	38	52.08	4.88	39	6.3%	-1.28 [-1.78, -0.79]	2018
Pasyar et al. 2020	38.43	10.16	30	39.53	10.15	30	6.2%	-0.11 [-0.61, 0.40]	2020
Yadegari et al. 2021	36.42	6.62	42	48.78	6.9	42	6.2%	-1.81 [-2.32, -1.30]	2021
Liao & Zhu 2022	46.72	2.433	36	47.05	2.013	36	6.4%	-0.15 [-0.61, 0.32]	2022
Ugras et al. 2023	25.1	6.5	43	29.7	6.2	43	6.5%	-0.72 [-1.15, -0.28]	2023
Abbasnia et al. 2023	-8.165	10.11	98	3.43	15.88	47	6.8%	-0.94 [-1.30, -0.58]	2023
**Subtotal (95% CI)**			**322**			**272**	**44.8%**	**-0.84 [-1.25, -0.42]**	
Heterogeneity: Tau² = 0.26; Chi² = 34.23, df = 6 (P < 0.00001); I² = 82%									
Test for overall effect: Z = 3.93 (P < 0.0001)									
**1.1.2 Preoperative Counseling**									
Lu & Zhang 2018	42.85	7.45	45	51.06	8.05	45	6.5%	-1.05 [-1.49, -0.61]	2018
Toğaç & Yılmaz 2021	42.79	4.28	62	50.98	5.45	62	6.6%	-1.66 [-2.07, -1.25]	2021
**Subtotal (95% CI)**			**107**			**107**	**13.1%**	**-1.36 [-1.96, -0.76]**	
Heterogeneity: Tau² = 0.14; Chi² = 3.95, df = 1 (P = 0.05); I² = 75%									
Test for overall effect: Z = 4.45 (P < 0.00001)									
**1.1.3 Information Provision**									
Lim et al. 2011	-8.6	23.2	56	-4.9	24.8	64	6.8%	-0.15 [-0.51, 0.21]	2011
Tou et al. 2012	44.3	13.6	16	41.4	15.2	15	5.3%	0.20 [-0.51, 0.90]	2012
Amini et al. 2018	32.35	8.85	40	42.05	15.24	20	6.0%	-0.84 [-1.40, -0.28]	2018
**Subtotal (95% CI)**			**112**			**99**	**18.1%**	**-0.28 [-0.82, 0.25]**	
Heterogeneity: Tau² = 0.15; Chi² = 6.13, df = 2 (P = 0.05); I² = 67%									
Test for overall effect: Z = 1.04 (P = 0.30)									
**1.1.4 Psychological Intervention**									
Garcia et al. 2018	36.3	10.1	25	36.2	11.2	25	6.0%	0.01 [-0.55, 0.56]	2018
Felix et al. 2018	43	3.54	12	46.83	3.76	12	4.6%	-1.01 [-1.87, -0.15]	2018
Bulut & Karabulut 2023	41.26	9.434	58	48.26	9.236	57	6.8%	-0.74 [-1.12, -0.37]	2023
Menevşe & Yayla 2024	18.79	6.38	56	28.82	6.3	56	6.6%	-1.57 [-2.00, -1.15]	2024
**Subtotal (95% CI)**			**151**			**150**	**24.0%**	**-0.83 [-1.51, -0.16]**	
Heterogeneity: Tau² = 0.39; Chi² = 20.60, df = 3 (P = 0.0001); I² = 85%									
Test for overall effect: Z = 2.43 (P = 0.02)									
**Total (95% CI)**			**692**			**628**	**100.0%**	**-0.80 [-1.10, -0.51]**	
Heterogeneity: Tau² = 0.30; Chi² = 93.56, df = 15 (P < 0.00001); I² = 84%									
Test for overall effect: Z = 5.35 (P < 0.00001)									
Test for subgroup differences: Chi² = 6.99, df = 3 (P = 0.07), I² = 57.1%									

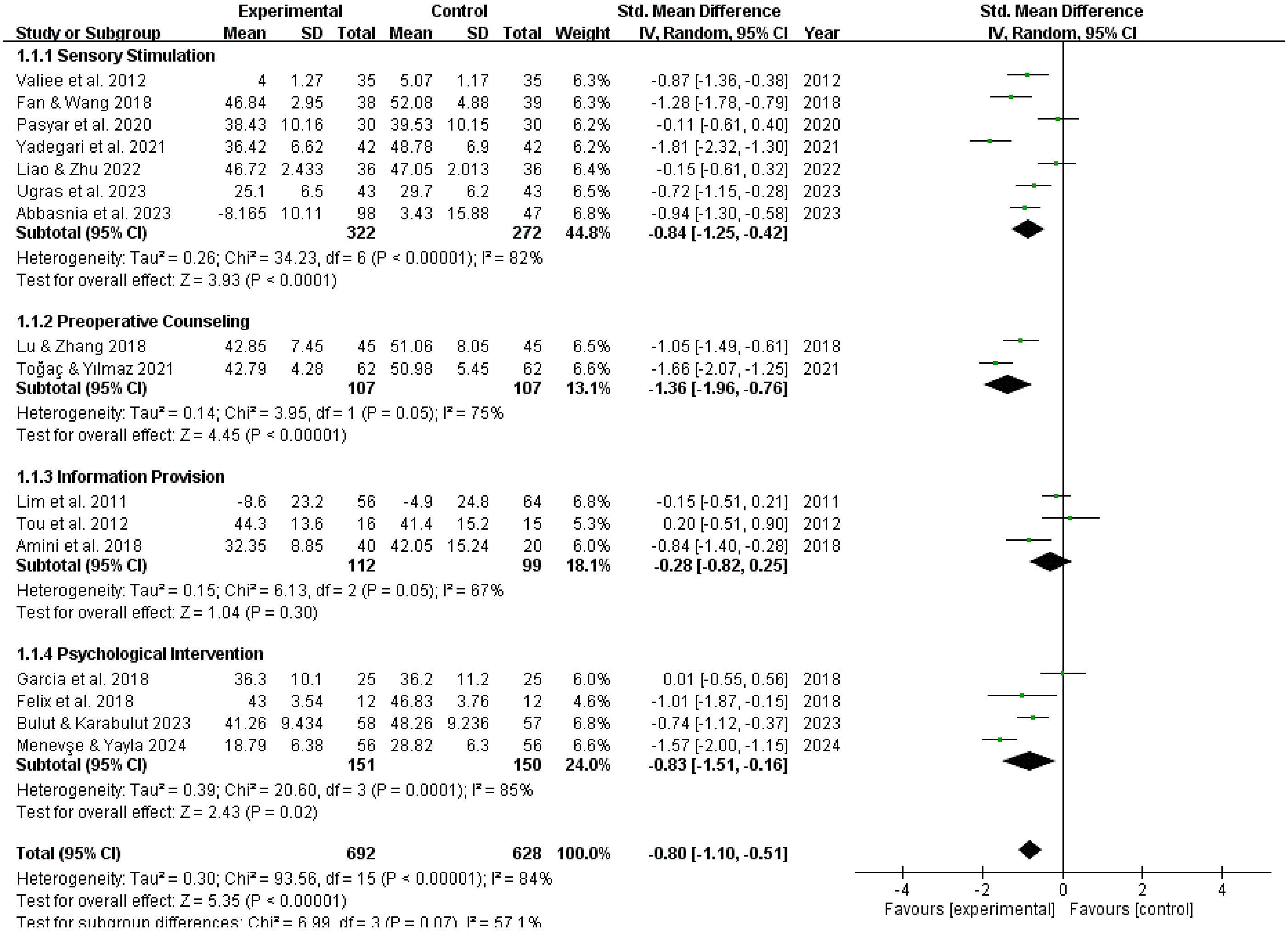

(b) Postoperative Anxiety Within Postoperative 24 Hours

Study or Subgroup	Experimental Mean	Experimental SD	Experimental Total	Control Mean	Control SD	Control Total	Weight	Std. Mean Difference IV, Random, 95% CI	Year
**2.1.1 Sensory Stimulation**									
Nilsson et al. 2005	0.25	0.76	50	1.1	2	25	11.1%	-0.64 [-1.14, -0.15]	2005
Akelma et al. 2020	38.22	6	58	41.05	4.22	59	12.6%	-0.54 [-0.91, -0.17]	2020
Soylu & Kartın 2021	36.26	6.37	26	36	7.09	27	10.5%	0.04 [-0.50, 0.58]	2021
**Subtotal (95% CI)**			**134**			**111**	**34.2%**	**-0.41 [-0.79, -0.03]**	
Heterogeneity: Tau² = 0.06; Chi² = 3.99, df = 2 (P = 0.14); I² = 50%									
Test for overall effect: Z = 2.14 (P = 0.03)									
**2.1.2 Preoperative Counseling**									
Toğaç & Yılmaz 2021	39.08	3.49	62	44.41	4.77	62	12.4%	-1.27 [-1.65, -0.88]	2021
Goktas et al. 2022	55.79	4.89	30	61.86	3.76	30	10.2%	-1.37 [-1.94, -0.81]	2022
**Subtotal (95% CI)**			**92**			**92**	**22.5%**	**-1.30 [-1.62, -0.98]**	
Heterogeneity: Tau² = 0.00; Chi² = 0.09, df = 1 (P = 0.76); I² = 0%									
Test for overall effect: Z = 7.98 (P < 0.00001)									
**2.1.3 Information Provision**									
Tou et al. 2012	36.2	8.2	16	36.4	9.3	15	8.6%	-0.02 [-0.73, 0.68]	2012
**Subtotal (95% CI)**			**16**			**15**	**8.6%**	**-0.02 [-0.73, 0.68]**	
Heterogeneity: Not applicable									
Test for overall effect: Z = 0.06 (P = 0.95)									
**2.1.4 Psychological Intervention**									
Hasanpour-Dehkordi et al. 2019	35.4	4.58	35	40.05	4.96	35	11.0%	-0.96 [-1.46, -0.47]	2019
Aktaş & İlgin 2023	38.7	9.78	60	46.8	6.13	30	11.5%	-0.92 [-1.38, -0.46]	2023
Bulut & Karabulut 2023	34.98	6.734	58	45.93	10.397	57	12.2%	-1.24 [-1.64, -0.84]	2023
**Subtotal (95% CI)**			**153**			**122**	**34.7%**	**-1.07 [-1.32, -0.81]**	
Heterogeneity: Tau² = 0.00; Chi² = 1.32, df = 2 (P = 0.52); I² = 0%									
Test for overall effect: Z = 8.10 (P < 0.00001)									
**Total (95% CI)**			**395**			**340**	**100.0%**	**-0.80 [-1.11, -0.49]**	
Heterogeneity: Tau² = 0.16; Chi² = 30.83, df = 8 (P = 0.0002); I² = 74%									
Test for overall effect: Z = 5.05 (P < 0.00001)									
Test for subgroup differences: Chi² = 19.93, df = 3 (P = 0.0002), I² = 85.0%									

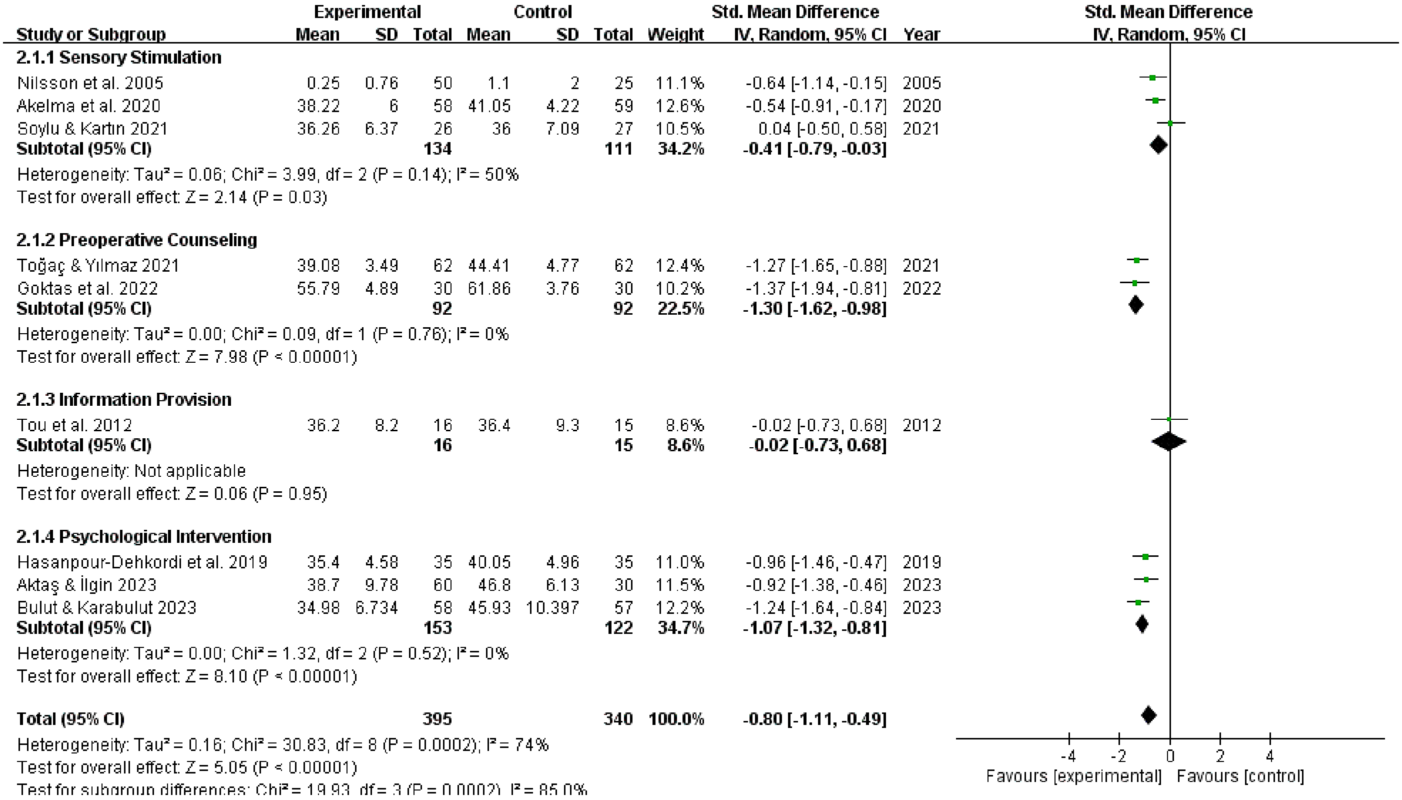

(c) Postoperative Pain Within Postoperative 24 Hours

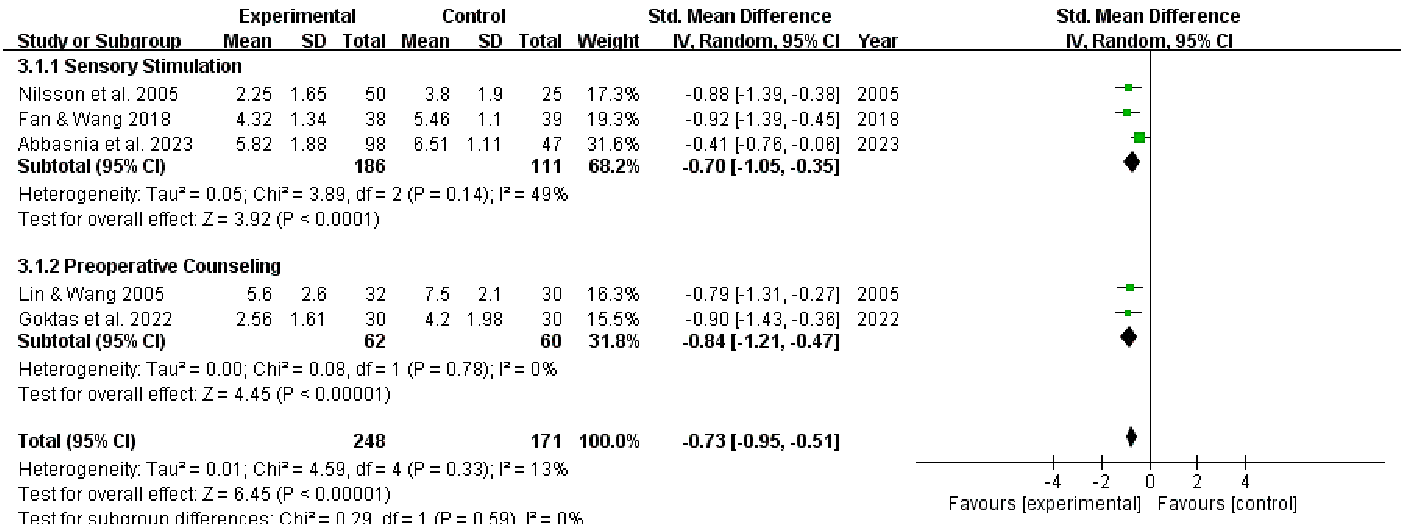

	Experimental			Control				Std. Mean Difference	
Study or Subgroup	Mean	SD	Total	Mean	SD	Total	Weight	IV, Random, 95% CI	Year
**3.1.1 Sensory Stimulation**									
Nilsson et al. 2005	2.25	1.65	50	3.8	1.9	25	17.3%	-0.88 [-1.39, -0.38]	2005
Fan & Wang 2018	4.32	1.34	38	5.46	1.1	39	19.3%	-0.92 [-1.39, -0.45]	2018
Abbasnia et al. 2023	5.82	1.88	98	6.51	1.11	47	31.6%	-0.41 [-0.76, -0.06]	2023
**Subtotal (95% CI)**			**186**			**111**	**68.2%**	**-0.70 [-1.05, -0.35]**	
Heterogeneity: Tau² = 0.05; Chi² = 3.89, df = 2 (P = 0.14); I² = 49%									
Test for overall effect: Z = 3.92 (P < 0.0001)									
**3.1.2 Preoperative Counseling**									
Lin & Wang 2005	5.6	2.6	32	7.5	2.1	30	16.3%	-0.79 [-1.31, -0.27]	2005
Goktas et al. 2022	2.56	1.61	30	4.2	1.98	30	15.5%	-0.90 [-1.43, -0.36]	2022
**Subtotal (95% CI)**			**62**			**60**	**31.8%**	**-0.84 [-1.21, -0.47]**	
Heterogeneity: Tau² = 0.00; Chi² = 0.08, df = 1 (P = 0.78); I² = 0%									
Test for overall effect: Z = 4.45 (P < 0.00001)									
**Total (95% CI)**			**248**			**171**	**100.0%**	**-0.73 [-0.95, -0.51]**	
Heterogeneity: Tau² = 0.01; Chi² = 4.59, df = 4 (P = 0.33); I² = 13%									
Test for overall effect: Z = 6.45 (P < 0.00001)									
Test for subgroup differences: Chi² = 0.29, df = 1 (P = 0.59), I² = 0%									

Figure S3: Forest plot of the effectiveness of non-pharmacological interventions among patients undergoing abdominal surgery on: (a) preoperative anxiety within preoperative 24 hours; (b) postoperative anxiety within postoperative 24 hours; and (c) postoperative pain within postoperative 24 hours.

(a) Preoperative Anxiety Within Preoperative 24 Hours

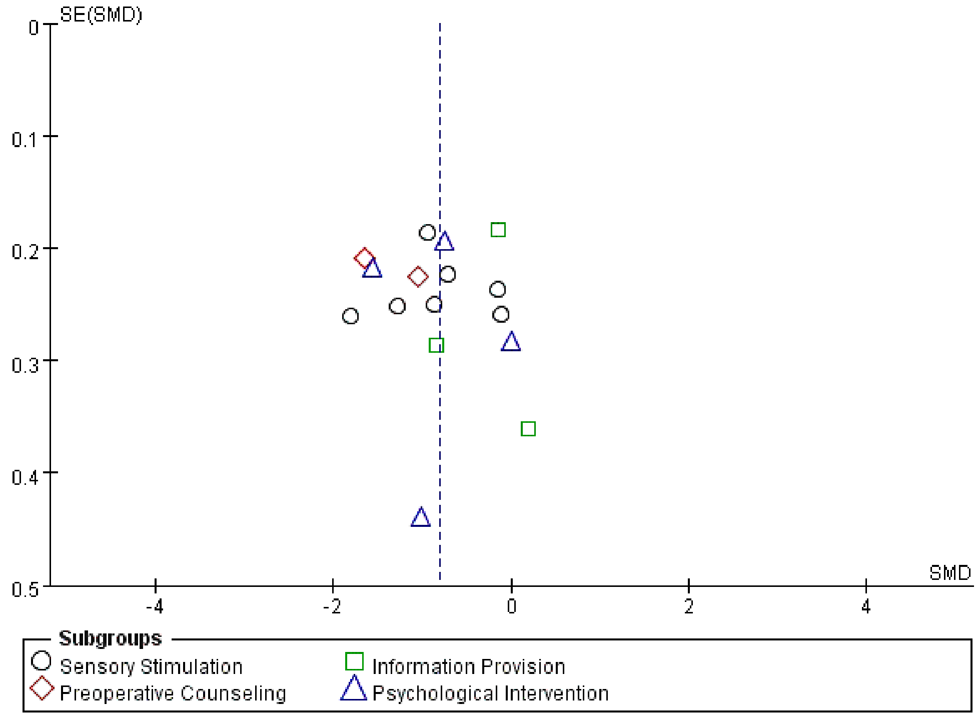

(b) Postoperative Anxiety Within Postoperative 24 Hours

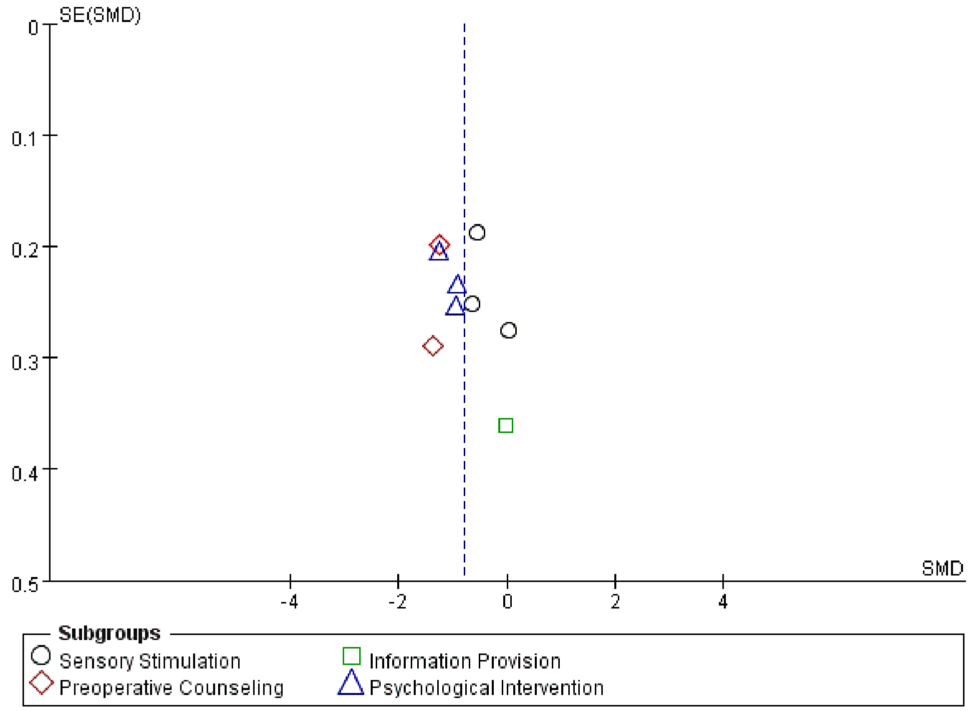

Figure S4: Funnel plots of the meta-analyses of: (a) preoperative anxiety within preoperative 24 hours; and (b) postoperative anxiety within postoperative 24 hours.
