# Supplementary material for: The Effectiveness of Non‐Pharmacological Interventions on Preoperative and Postoperative Anxiety Among Patients Undergoing Abdominal Surgery: A Systematic Review and Meta‐Analysis
Source: Worldviews Evid Based Nurs. 2026 Feb 18;23(1):e70099. doi: 10.1111/wvn.70099 (PMC12917301; doi:10.1111/wvn.70099)
Supplement: Supplementary file 4 — Table S1: Identified keywords in PICOS form. Table S2: Search strategy and initial results. Table S3: Summary table of the characteristics of the included studies. [file WVN-23-0-s002.docx]

| Table S1. Identified Keywords in PICOS Form | |
| --- | --- |
| Participant (P) | ‘abdominal surgery’ or ‘abdominal operation’ or ‘gastrointestinal’ or ‘gastrectomy’ or ‘appendectomy’ or ‘laparotomy’ or ‘cholecystectomy’ or ‘hepatectomy’ or ‘Whipple’ or ‘bowel resection’ or ‘colectomy’ or ‘patch repair’ or ‘wedge resection’ or ‘hernia repair’ or ‘Herniorrhaphy’ or ‘splenectomy’ or ‘pancreatectomy’ or ‘anterior resection’ or ‘ileostomy’ or ‘colostomy’ or ‘stoma’ or ‘esophagectomy’ or ‘colorectal’ or ‘hepatobiliary’ or ‘bariatric’ or ‘gallbladder’ or ‘pancreas’ or ‘stomach’ or ‘colon’ or ‘small bowel’ or ‘large bowel’ or ‘liver’ or ‘bile duct’ or ‘pelvic exenteration’ |
| Intervention (I) | ‘supplementary’ or ‘non-pharmacological’ or ‘virtual reality’ or ‘cognitive behavioral therapy’ or ‘music therapy’ or ‘aromatherapy’ or ‘education’ or ‘psychological intervention’ or ‘complementary’ or ‘massage’ or ‘relaxation technique’ or ‘relaxation therapy’ or ‘coping strategies’ or ‘hypnosis’ or ‘breathing exercise’ or ‘meditation’ or ‘mindfulness’ or ‘reflexology’ or ‘distraction’ or ‘acupressure’ |
| Outcome (O) | ‘anxiety’ or ‘anxious’ |
| Study Format (S) | ‘Randomized controlled trials’ |

| **Table S2: Search strategy and initial results** | | |
| --- | --- | --- |
|  | MEDLINE(R) 1987 to March 1, 2024 (OVID platform) |  |
| 1 | (((General Surgery/) AND (Abdomen/ or gallbladder/ or intestines/ or colon/ or Colonic Diseases/ or gastrointestinal tract/ or Gastrointestinal Neoplasms/ or Upper Gastrointestinal Tract/ or Lower Gastrointestinal Tract/ or pancreas/ or Pancreas Transplantation/ or Pancreatic Neoplasms/ or bariatrics/ or stomach/ or Intestine, Small/ or Intestine, Large/ or liver/ or Liver Diseases/ or Liver Transplantation/ or Liver Neoplasms/ or spleen/ or Splenic Diseases/ or Splenic Neoplasms/ or Bile Ducts/ or Colorectal Neoplasms/ or hernia/ or abdominal hernia/ or Esophageal neoplasms/ or Esophagus/ or Esophageal Diseases/ or Digestive System Diseases/ or Rectal Neoplasms/ or Rectal Diseases/ or Biliary Tract Diseases/)) OR (Colorectal Surgery/ or gastrectomy/ or Appendectomy/ or Laparotomy/ or Cholecystectomy/ or Cholecystectomy, Laparoscopic/ or Hepatectomy/ or Colectomy/ or Herniorrhaphy/ or Splenectomy/ or pancreatectomy/ or Ileostomy/ or Colostomy/ or Esophagectomy/ or Pelvic Exenteration/)) | 232401 |
| 2 | limit 1 to (full text and yr="1987 -Current") | 59910 |
| 3 | (((Abdom* or pancrea* or hepat* or colo* or bariatric or gallbladder or bowel or stomach or gastrointestinal or duct or liver or esophagi* or sple*) and (surger* or operation*)) or (gastrectomy or appendectomy or laparotomy or hepatectomy or whipple or colectomy or wedge resection or Herniorrhaphy or splenectomy or cholecystectomy or bowel resection or patch repair or pancreatectomy or wedge resection or hernia repair or anterior resection or ileostomy or colostomy or stoma or esophagectomy or pelvic exenteration)).mp. | 782031 |
| 4 | limit 3 to (full text and yr="1987 -Current") | 202999 |
| 5 | #2 OR #4 | 203023 |
| 6 | Cognitive Behavioral Therapy/ OR Relaxation Therapy/ or Complementary Therapies/ or Meditation/ or Psychosocial Intervention/ or Aromatherapy/ or Music Therapy/ or Massage/ or Adaptation, Psychological/ or Virtual Reality/ or Breathing Exercises/ or Hypnosis/ or education/ or health education/ or mindfulness/ or Musculoskeletal Manipulations/ or acupressure/ or acupuncture therapy/ | 300364 |
| 7 | limit 6 to (full text and yr="1987 -Current") | 81954 |
| 8 | (((cogniti* behavior* OR nature or music or aroma* or relaxation or hypno) adj3 (therap*)) or (relaxation technique or coping strateg* or complement* or supplement* or non pharmacolog* or Meditat* or psycholog* Intervention or education or Massage or Virtual Reality or hypno* or breathing exercise* or mindfulness or reflexology or distraction or acupressure or acupuncture)).mp. | 2008765 |
| 9 | limit 8 to (full text and yr="1987 -Current") | 580047 |
| 10 | #7 OR #9 | 605001 |
| 11 | Anxiety/ | 99976 |
| 12 | limit 11 to (full text and yr="1987 -Current") | 31050 |
| 13 | (anxi* or worr* or nervous* or fear*).mp. | 930346 |
| 14 | limit 13 to (full text and yr="1987 -Current") | 248172 |
| 15 | #12 OR #14 | 248172 |
| 16 | Randomized Controlled Trials as Topic/ | 167286 |
| 17 | limit 16 to (full text and yr="1987 -Current") | 65136 |
| 18 | ((randomi* clinical trial*) OR (randomi* controlled clinical trial*) OR (randomi* clinical controlled trial*) OR (trial*, randomi* clinical)).mp. | 75875 |
| 19 | limit 18 to (full text and yr="1987 -Current") | 26362 |
| 20 | #17 OR #19 | 85753 |
| 21 | #5 AND #10 AND #15 AND #20 | 19 |
|  | AMED (Allied and Complementary Medicine) 1987 to March 1, 2024 (OVID platform) |  |
| 1 | ((((Surgery/) or (Surgery operative/)) AND (Abdomen/ or gallbladder/ or intestines/ or colon/ or gastrointestinal tract/ or pancreas/ or stomach/ or liver/ or Bile Ducts/ or Colorectal neoplasms/ or Pancreatic neoplasms/ or hernia/ or Splenic disease/ or Gastrointestinal neoplasms/ or Colonic disease/ or Esophageal neoplasms/ or Liver disease/ or Digestive system disease/ or Rectal neoplasms/ or Rectal disease/ or Biliary tract disease/)) OR (gastrectomy/ or Laparotomy/)) | 407 |
| 2 | limit 1 to (full text and yr="1987 -Current") | 103 |
| 3 | (((Abdom* OR pancrea* OR hepat* OR colo* OR bariatric OR gallbladder OR bowel OR stomach OR gastrointestinal OR duct OR liver OR esophagi* OR sple*) AND (surger* OR operation*)) OR (gastrectomy or appendectomy or laparotomy or hepatectomy or whipple or colectomy or wedge resection or Herniorrhaphy or splenectomy or cholecystectomy or bowel resection or patch repair or pancreatectomy or wedge resection or hernia repair or anterior resection or ileostomy or colostomy or stoma or esophagectomy or pelvic exenteration)).mp. | 1246 |
| 4 | limit 3 to (full text and yr="1987 -Current") | 345 |
| 5 | #2 OR #4 | 364 |
| 6 | Cognitive therapy/ OR Behavior therapy/ OR Relaxation/ or Complementary Therapies/ or therapy/ or Meditation/ or Psychotherapy/ or Aroma therapy/ or Music Therapy/ or Adaptation psychological/ or Virtual Reality/ or Breathing Exercises/ or Hypnosis/ or education/ or Musculoskeletal Manipulations/ or massage/ or acupressure/ or acupuncture therapy/ or reflexology/ | 70297 |
| 7 | limit 6 to (full text and yr="1987 -Current") | 16225 |
| 8 | (((cogniti* behavior* OR nature or music or aroma* or relaxation or hypno) adj3 (therap*)) or (relaxation technique or coping strateg* or complement* or supplement* or non pharmacolog* or Meditat* or psycholog* Intervention or education or Massage or Virtual Reality or hypno* or breathing exercise* or mindfulness or reflexology or distraction or acupressure or acupuncture)).mp. | 61291 |
| 9 | limit 8 to (full text and yr="1987 -Current") | 14749 |
| 10 | #7 OR #9 | 23717 |
| 11 | Anxiety/ | 1359 |
| 12 | limit 11 to (full text and yr="1987 -Current") | 379 |
| 13 | (anxi* or worr* or nervous* or fear*).mp. | 15611 |
| 14 | limit 13 to (full text and yr="1987 -Current") | 4630 |
| 15 | #12 OR #14 | 4630 |
| 16 | Randomized Controlled Trials/ | 3211 |
| 17 | limit 16 to (full text and yr="1987 -Current") | 1403 |
| 18 | ((randomi* clinical trial*) OR (randomi* controlled clinical trial*) OR (randomi* clinical controlled trial*) OR (trial*, randomi* clinical)).mp. | 2328 |
| 19 | limit 18 to (full text and yr="1987 -Current") | 764 |
| 20 | #17 OR #19 | 2037 |
| 21 | #5 AND #10 AND #15 AND #20 | 1 |
|  | Ovid Nursing Database 1987to March 1, Week 1 2024 (OVID platform) |  |
| 1 | (((Operative Surgery/) AND (Abdomen/ or gallbladder/ or Gallbladder Diseases/ or Gallbladder Neoplasms/ or intestines/ or colon/ or pancreas/ or pancreas transplantation/ or bariatrics/ or stomach/ or Stomach Diseases/ or Stomach Neoplasms/ or small Intestine/ or Large Intestine/ or liver/ or Liver Diseases/ or Liver Neoplasms/ or Liver Transplantation/ or Bile Ducts/ or spleen/ or Esophageal Diseases/ or Esophageal Neoplasms/or hernia/ or Rectal Neoplasms/ or Colorectal Neoplasms/)) OR (Colorectal Surgery/ or gastrectomy/ or Appendectomy/ or Laparoscopy/ or Laparotomy/ or Cholecystectomy/ or Laparoscopic Cholecystectomy / or Colectomy/ or Splenectomy/ or pancreatectomy/ or Ileostomy/ or Ostomy/ or Colostomy/ or Pelvic Exenteration/)) | 1855 |
| 2 | limit 1 to (full text and yr="1987 -Current") | 1195 |
| 3 | (((Abdom* OR pancrea* OR hepat* OR colo* OR bariatric OR gallbladder OR bowel OR stomach OR gastrointestinal OR duct OR liver OR esophagi* OR sple*) AND (surger* OR operation*)) OR (gastrectomy or appendectomy or laparotomy or hepatectomy or whipple or colectomy or wedge resection or Herniorrhaphy or splenectomy or cholecystectomy or bowel resection or patch repair or pancreatectomy or wedge resection or hernia repair or anterior resection or ileostomy or colostomy or stoma or esophagectomy or pelvic exenteration)).mp. | 3339 |
| 4 | limit 3 to (full text and yr="1987 -Current") | 2166 |
| 5 | #2 OR #4 | 2382 |
| 6 | Perioperative Nursing/ OR Cognitive Therapy/ OR Behavior Therapy/ OR Relaxation Techniques/ or Alternative Therapies/ or Meditation/ or Psychotherapy/ or Aromatherapy/ or Music Therapy/ or Massage/ or Coping/ or Psychological Adaptation/ or Virtual Reality/ or Breathing Exercises/ or Hypnosis/ or education/ or Health Education/ or Preoperative Education/ or acupressure/ or acupuncture/ or Mindfulness-Based Stress Reduction/ or distraction/ | 30873 |
| 7 | limit 6 to (full text and yr="1987 -Current") | 12689 |
| 8 | (((cogniti* behavior* OR nature or music or aroma* or relaxation or hypno) adj3 (therap*)) or (relaxation technique or coping strateg* or complement* or supplement* or non pharmacolog* or Meditat* or psycholog* Intervention or education or Massage or Virtual Reality or hypno* or breathing exercise* or mindfulness or reflexology or distraction or acupressure or acupuncture)).mp. | 44423 |
| 9 | limit 8 to (full text and yr="1987 -Current") | 21226 |
| 10 | #7 OR #9 | 23055 |
| 11 | Anxiety/ | 6798 |
| 12 | limit 11 to (full text and yr="1987 -Current") | 3159 |
| 13 | (anxi* or worr* or nervous* or fear*).mp. | 11634 |
| 14 | limit 13 to (full text and yr="1987 -Current") | 5698 |
| 15 | #12 OR #14 | 5698 |
| 16 | Clinical Trials/ | 11288 |
| 17 | limit 16 to (full text and yr="1987 -Current") | 6033 |
| 18 | ((randomi* clinical trial*) OR (randomi* controlled clinical trial*) OR (randomi* clinical controlled trial*) OR (trial*, randomi* clinical)).mp. | 1505 |
| 19 | limit 18 to (full text and yr="1987 -Current") | 780 |
| 20 | #17 OR #19 | 6122 |
| 21 | #5 AND #10 AND #15 AND #20 | 3 |
|  | APA PsycInfo 1987 to March 1, Week 1 2024 (OVID platform) |  |
| 1 | (((Surgery/) AND (Abdomen/ or Gastrointestinal System/ or intestines/ or Colon Disorders/ or pancreas/ or Organ Transplantation/ or stomach/ or liver/ or Liver Disorders/ or spleen/ or Esophagus/ or Neoplasms/)) OR (Bariatric Surgery/ or Colostomy/)) | 3029 |
| 2 | limit 1 to (full text and yr="1987 -Current") | 1077 |
| 3 | (((Abdom* OR pancrea* OR hepat* OR colo* OR bariatric OR gallbladder OR bowel OR stomach OR gastrointestinal OR duct OR liver OR esophagi* OR sple*) AND (surger* OR operation*)) OR (gastrectomy or appendectomy or laparotomy or hepatectomy or whipple or colectomy or wedge resection or Herniorrhaphy or splenectomy or cholecystectomy or bowel resection or patch repair or pancreatectomy or wedge resection or hernia repair or anterior resection or ileostomy or colostomy or stoma or esophagectomy or pelvic exenteration)).mp. | 7892 |
| 4 | limit 3 to (full text and yr="1987 -Current") | 2248 |
| 5 | #2 OR #4 | 2620 |
| 6 | Educational Programs/ OR Cognitive Behavior Therapy/ or Cognitive Therapy/ OR Behavior Therapy/ OR Relaxation Therapy/ or relaxation/ or yoga/ or Progressive Relaxation Therapy/ or anxiety management/ or Muscle Relaxation/ or Meditation/ or Psychotherapy/ or Aromatherapy/ or Music Therapy/ or Massage/ or Coping Behavior/ or Guided Imagery/ or Virtual Reality/ or Hypnosis/ or education/ or Psychology Education/ or Health Education/ or acupuncture/ or Mindfulness-Based Interventions/ or Mindfulness/ or distraction/ | 318221 |
| 7 | limit 6 to (full text and yr="1987 -Current") | 58359 |
| 8 | (((cogniti* behavior* OR nature or music or aroma* or relaxation or hypno) adj3 (therap*)) or (relaxation technique or coping strateg* or complement* or supplement* or non pharmacolog* or Meditat* or psycholog* Intervention or education or Massage or Virtual Reality or hypno* or breathing exercise* or mindfulness or reflexology or distraction or acupressure or acupuncture)).mp. | 833213 |
| 9 | limit 8 to (full text and yr="1987 -Current") | 150985 |
| 10 | #7 OR #9 | 171887 |
| 11 | Anxiety/ | 80375 |
| 12 | limit 11 to (full text and yr="1987 -Current") | 17741 |
| 13 | (anxi* or worr* or nervous* or fear*).mp. | 516101 |
| 14 | limit 13 to (full text and yr="1987 -Current") | 114367 |
| 15 | #12 OR #14 | 114367 |
| 16 | Randomized Controlled Trials/ | 1058 |
| 17 | limit 16 to (full text and yr="1987 -Current") | 370 |
| 18 | ((randomi* clinical trial*) OR (randomi* controlled clinical trial*) OR (randomi* clinical controlled trial*) OR (trial*, randomi* clinical)).mp. | 10462 |
| 19 | limit 18 to (full text and yr="1987 -Current") | 3742 |
| 20 | #17 OR #19 | 4094 |
| 21 | #5 AND #10 AND #15 AND #20 | 0 |
|  | Embase 1987 to March 1, 2024 (OVID platform) |  |
| 1 | (abdominal surgery/ or bariatric surgery/ or colorectal surgery/ or general surgery/ or intestine surgery/or laparoscopic surgery/ or major surgery/or open surgery/or biliary tract surgery/ or colon surgery/ or esophagus surgery/ or gastric bypass surgery/ or gastrointestinal surgery/ or liver surgery/ or rectum surgery/ or stomach surgery/ or pancreas surgery/) | 288844 |
| 2 | limit 1 to (full text and yr="1987 -Current") | 84587 |
| 3 | (((Abdom* OR pancrea* OR hepat* OR colo* OR bariatric OR gallbladder OR bowel OR stomach OR gastrointestinal OR duct OR liver OR esophagi* OR sple*) AND (surger* OR operation*)) OR (gastrectomy or appendectomy or laparotomy or hepatectomy or whipple or colectomy or wedge resection or Herniorrhaphy or splenectomy or cholecystectomy or bowel resection or patch repair or pancreatectomy or wedge resection or hernia repair or anterior resection or ileostomy or colostomy or stoma or esophagectomy or pelvic exenteration)).mp. | 1429953 |
| 4 | limit 3 to (full text and yr="1987 -Current") | 381374 |
| 5 | #2 OR #4 | 400179 |
| 6 | Cognitive Behavioral Therapy/ OR behavior therapy/ OR cognitive therapy/ OR Music Therapy/ OR Aromatherapy/ OR education/ OR patient education/ OR preoperative education/ OR health education/ OR psychotherapy/ OR alternative medicine/ OR massage/ OR relaxation training/ OR coping behavior/ OR hypnosis/ OR breathing exercise/ OR Meditation/ OR Mindfulness/ OR Mindfulness-based stress reduction/ OR Mindfulness meditation/ OR reflexology/ OR acupressure/ OR acupuncture/ | 1097366 |
| 7 | limit 6 to (full text and yr="1987 -Current") | 276109 |
| 8 | (((cogniti* behavior* OR nature or music or aroma* or relaxation or hypno) adj3 (therap*)) or (relaxation technique or coping strateg* or complement* or supplement* or non pharmacolog* or Meditat* or psycholog* Intervention or education or Massage or Virtual Reality or hypno* or breathing exercise* or mindfulness or reflexology or distraction or acupressure or acupuncture)).mp. | 3009037 |
| 9 | limit 8 to (full text and yr="1987 -Current") | 786344 |
| 10 | #7 OR #9 | 836931 |
| 11 | Anxiety/ | 298120 |
| 12 | limit 11 to (full text and yr="1987 -Current") | 76149 |
| 13 | (anxi* or worr* or nervous* or fear*).mp. | 1835859 |
| 14 | limit 13 to (full text and yr="1987 -Current") | 363480 |
| 15 | #12 OR #14 | 363480 |
| 16 | "randomized controlled trial (topic)"/ | 269906 |
| 17 | limit 16 to (full text and yr="1987 -Current") | 82257 |
| 18 | ((randomi* clinical trial*) OR (randomi* controlled clinical trial*) OR (randomi* clinical controlled trial*) OR (trial*, randomi* clinical)).mp. | 119432 |
| 19 | limit 18 to (full text and yr="1987 -Current") | 34150 |
| 20 | #17 OR #19 | 110903 |
| 21 | #5 AND #10 AND #15 AND #20 | 64 |
|  | CINAHL Ultimate 1987 to March 1, Week 1 2024 (EBSCO Platform) |  |
| 1 | (((MH “Surgery, Operative”) AND ((MH “Abdomen”) or (MH “gallbladder”) or (MH “Gallbladder Neoplasms”) or (MH “Gallbladder diseases”) or (MH “Acalculous Cholecystitis”) or (MH “intestines”) or (MH “intestines Neoplasms”) or (MH “intestines, large”) or (MH “intestines, small”) or (MH “colon”) or (MH “Colonic Diseases”) or (MH “Colonic Neoplasms”) or (MH “Gastrointestinal Neoplasms”) or (MH ”pancreas”) or (MH “Pancreas Transplantation”) or (MH “stomach”) or (MH “stomach Neoplasms”) or (MH “stomach Diseases”) or (MH “liver”) or (MH “Liver Diseases”) or (MH “Liver Transplantation”) or (MH “Liver Neoplasms”) or (MH “spleen”) or (MH “Splenic Neoplasms”) or (MH “Bile Ducts”) or (MH “Bile Duct Neoplasms”) or (MH “Bile Duct Diseases”) or (MH “Colorectal Neoplasms”) or (MH “hernia”) or (MH “hernia, abdominal) or (MH “Esophagus”) or (MH “Esophageal neoplasms”) or (MH ”Esophageal Diseases”) or (MH ” Digestive System”) or (MH “Digestive System Diseases”) or (MH “Digestive System Neoplasms”) or (MH “rectum”) or (MH “Rectal Neoplasms”) or (MH “Rectal Diseases”) or (MH “Biliary Tract”) or (MH “Biliary Tract Neoplasms”) or (MH “Biliary Tract Diseases”)) OR ((MH “Bariatric Surgery”) or (MH “gastrectomy”) or (MH “Appendectomy”) or (MH “Laparotomy”) or (MH “Cholecystectomy”) or (MH “Cholecystectomy, Laparoscopic”) or (MH “Hepatectomy”) or (MH “Colectomy”) or (MH “Herniorrhaphy”) or (MH “Splenectomy”) or (MH “pancreatectomy”) or (MH “Surgical Stoma”) or (MH “Ileostomy”) or (MH “Colostomy”) or (MH “Pelvic Exenteration”) or (MH “Surgery, Laparoscopic”) or (MH “Pancreaticoduodenectomy”))) - Limiters - Full Text; Publication Date: 19870101-20240331 | 184 |
| 2 | TI (((Abdom* OR pancrea* OR hepat* OR colo* OR bariatric OR gallbladder OR bowel OR stomach OR gastrointestinal OR duct OR liver OR esophagi* OR sple*) AND (surger* OR operation*)) OR (gastrectomy or appendectomy or laparotomy or hepatectomy or whipple or colectomy or wedge resection or Herniorrhaphy or splenectomy or cholecystectomy or bowel resection or patch repair or pancreatectomy or wedge resection or hernia repair or anterior resection or ileostomy or colostomy or stoma or esophagectomy or pelvic exenteration)) - Limiters - Full Text; Publication Date: 19870101-20240331 | 4185 |
| 3 | AB (((Abdom* OR pancrea* OR hepat* OR colo* OR bariatric OR gallbladder OR bowel OR stomach OR gastrointestinal OR duct OR liver OR esophagi* OR sple*) AND (surger* OR operation*)) OR (gastrectomy or appendectomy or laparotomy or hepatectomy or whipple or colectomy or wedge resection or Herniorrhaphy or splenectomy or cholecystectomy or bowel resection or patch repair or pancreatectomy or wedge resection or hernia repair or anterior resection or ileostomy or colostomy or stoma or esophagectomy or pelvic exenteration)) - Limiters - Full Text; Publication Date: 19870101-20240331 | 9028 |
| 4 | S1 or S2 or S3 | 11129 |
| 5 | ((MH “virtual reality”) or (MH “Cognitive Therapy”) or (MH “Behavior Therapy”) or (MH “music therapy”) or (MH “aromatherapy”) or (MH “education”) or (MH “health education”) or (MH “preoperative education”) or (MH ”Psychosocial Intervention”) or (MH “Alternative Therapies”) or (MH “massage”) or (MH “relaxation techniques”) or (MH “coping”) or (MH “hypnosis”) or (MH “breathing exercises”) or (MH “meditation”) or (MH “mindfulness”) or (MH “yoga”) or (MH “reflexology”) or (MH “distraction”) or (MH “acupressure”) or (MH “Acupuncture”)) - Limiters - Full Text; Publication Date: 19870101-20240331 | 73724 |
| 6 | TI (((cogniti* behavior* OR nature or music or aroma* or relaxation or hypno) adj3 (therap*)) or (relaxation technique or coping strateg* or complement* or supplement* or non pharmacolog* or Meditat* or psycholog* Intervention or education or Massage or Virtual Reality or hypno* or breathing exercise* or mindfulness or reflexology or distraction or acupressure or acupuncture)) - Limiters - Full Text; Publication Date: 19870101-20240331 | 59041 |
| 7 | AB (((cogniti* behavior* OR nature or music or aroma* or relaxation or hypno) adj3 (therap*)) or (relaxation technique or coping strateg* or complement* or supplement* or non pharmacolog* or Meditat* or psycholog* Intervention or education or Massage or Virtual Reality or hypno* or breathing exercise* or mindfulness or reflexology or distraction or acupressure or acupuncture)) - Limiters - Full Text; Publication Date: 19870101-20240331 | 122979 |
| 8 | S5 OR S6 OR S7 | 205195 |
| 9 | (MH “Anxiety”) - Limiters - Full Text; Publication Date: 19870101-20240331 | 18999 |
| 10 | TI (anxi* or worr* or nervous* or fear*) - Limiters - Full Text; Publication Date: 19870101-20240331 | 13827 |
| 11 | AB (anxi* or worr* or nervous* or fear*) - Limiters - Full Text; Publication Date: 19870101-20240331 | 43821 |
| 12 | S9 OR S10 OR S11 | 55493 |
| 13 | (MH “Randomized Controlled Trials”) - Limiters - Full Text; Publication Date: 19870101-20240331 | 27381 |
| 14 | TI ((randomi* clinical trial*) OR (randomi* controlled clinical trial*) OR (randomi* clinical controlled trial*) OR (trial*, randomi* clinical)) - Limiters - Full Text; Publication Date: 19870101-20240331 | 2795 |
| 15 | AB ((randomi* clinical trial*) OR (randomi* controlled clinical trial*) OR (randomi* clinical controlled trial*) OR (trial*, randomi* clinical)) - Limiters - Full Text; Publication Date: 19870101-20240331 | 5057 |
| 16 | S13 OR S14 OR S15 | 31071 |
| 17 | S4 AND S8 AND S12 AND S16 | 17 |
|  | Cochrane Library 1996-present |  |
| 1 | ((adult) and (((Abdom* OR pancrea* OR hepat* OR colo* OR bariatric OR gallbladder OR bowel OR stomach OR gastrointestinal OR duct OR liver OR esophagi* OR sple*) AND (surger* OR operation*)) OR (gastrectomy or appendectomy or laparotomy or hepatectomy or whipple or colectomy or wedge resection or Herniorrhaphy or splenectomy or cholecystectomy or bowel resection or patch repair or pancreatectomy or wedge resection or hernia repair or anterior resection or ileostomy or colostomy or stoma or esophagectomy or pelvic exenteration))):ab,ti,kw | 36104 |
| 2 | ((([mh "Specialties, Surgical"]) AND ([mh Abdomen] or [mh "Digestive System"] or [mh "Digestive System Diseases"] or [mh "Digestive System Neoplasms"] or [mh spleen] or [mh "Splenic Neoplasms"] or [mh hernia])) OR ([mh "Digestive System Surgical Procedures"] or [mh Transplantation] or [mh "Bariatric Surgery"] or [mh Laparotomy] or [mh Herniorrhaphy] or [mh Splenectomy] or [mh "Pelvic Exenteration"] or [mh Laparoscopes] or [mh "Colorectal Surgery"])) AND [mh adult] | 27744 |
| 3 | #1 OR #2 | 54606 |
| 4 | (((cogniti* behavior* OR nature or music or aroma* or relaxation or hypno) adj3 (therap*)) or (relaxation technique or coping strateg* or complement* or supplement* or non pharmacolog* or Meditat* or psycholog* Intervention or education or Massage or Virtual Reality or hypno* or breathing exercise* or mindfulness or reflexology or distraction or acupressure or acupuncture)):ab,ti,kw | 307095 |
| 5 | ([mh "Virtual reality"] or [mh Psychotherapy] or [mh "Complementary Therapies"] or [mh "Health Education"] or [mh "Musculoskeletal Manipulations"] or [mh "Adaptation, Psychological"] or [mh mindfulness] or [mh Acupuncture]) | 84137 |
| 6 | #4 OR #5 | 340596 |
| 7 | (anxi* or worr* or nervous* or fear*):ab,ti,kw | 118264 |
| 8 | [mh anxiety] | 12477 |
| 9 | #7 OR #8 | 118426 |
| 10 | ((randomi* clinical trial*) OR (randomi* controlled clinical trial*) OR (randomi* clinical controlled trial*) OR (trial*, randomi* clinical)):ab,ti,kw | 687712 |
| 11 | [mh "Randomized Controlled Trials as Topic"] | 54788 |
| 12 | #10 OR #11 | 698878 |
| 13 | #3 AND #6 AND #9 AND #12 | 300 |
|  | HyRead 1974 - present |  |
| 1 | (((((Abdom* OR pancrea* OR hepat* OR colo* OR bariatric OR gallbladder OR bowel OR stomach OR gastrointestinal OR duct OR liver OR esophagi* OR sple*) AND (surger* OR operation*)) OR (gastrectomy or appendectomy or laparotomy or hepatectomy or whipple or colectomy or wedge resection or Herniorrhaphy or splenectomy or cholecystectomy or bowel resection or patch repair or pancreatectomy or wedge resection or hernia repair or anterior resection or ileostomy or colostomy or stoma or esophagectomy or pelvic exenteration))) –全文檢索) AND (((((cogniti* behavior* OR nature or music or aroma* or relaxation or hypno) adj3 (therap*)) or (relaxation technique or coping strateg* or complement* or supplement* or non pharmacolog* or Meditat* or psycholog* Intervention or education or Massage or Virtual Reality or hypno* or breathing exercise* or mindfulness or reflexology or distraction or acupressure or acupuncture))) –全文檢索) AND ((anxiety) –全文檢索) | 0 |
|  | WANFANG DATA |  |
| 1 | ((腹部手术 OR 胃切除 OR 阑尾切除 OR 剖腹 OR 胆囊切除 OR 肝切除 OR 肠切除 OR 楔形切除 OR 胰腺切除 OR 疝修补 OR 脾切除 OR 前切除 OR 食管切除) - 全部) AND ((补充 OR 非药物 OR 虚拟 OR 认知 OR 音乐 OR 芳香 OR 教育 OR 干预 OR 按摩 OR 放松 OR 催眠 OR 呼吸 OR 冥想 OR 正念 OR 分心 OR 穴位) - 全部) AND ((焦虑) -全部) AND ((随机 AND 对照) - 全部) | 362 |

| **Table S3. Summary table of the characteristics of the included studies** | | | | | | |
| --- | --- | --- | --- | --- | --- | --- |
| Study  (Year);  Country | Categories | Type of surgery | Sample size (Intervention: Control) | Intervention groups  (Content and Dose (Time, Frequency, Period)) | Control groups | Outcomes (Instruments) |
| ***Anxiety measuring time: Before surgery*** | | | | | | |
| Valiee et al.  (2012).  Iran. | Sensory Stimulations (Tactile) | Elective abdominal surgery | 35:35 | **Content:** Acupressure via (1) a plastic bead was placed on the Shen men point of the non-prevailing ear, and it was left there to induce pressure by itself, and (2) immediately, the Yin tang point was pressured for 10 minutes using the thumb in a rotating manner with a mean of 20 to 25 cycles per minute.  **Dose:** 10 minutes, before surgery, once only. | Placebo to sham points (the external corner of the left eyebrow and the entrance of the cavity of the non-prevailing ear) and applying pressure by thumb for 10 minutes in a rotating manner for 20 to 25 cycles per minute. | Anxiety (Visual analogue Scale):   - Significantly lower in the IG than in the CG within preoperative 24 hours (Cohen's d = -0.87, *p* < 0.001). |
| Fan & Wang.  (2018).  China. | Sensory Stimulations (Tactile) | Elective laparoscopic cholecystectomy | 38:39 | **Content:** Usual care and intervention: pestle therapy with various steps, including (1) assess the skin condition and disinfect the pestle needle. Apply lifting technique with a “Wu Xing San Tai” pestle on “He Che Yin Nao” Segment for 4 minutes, (2) apply tapping technique with a "Wu Xing San Tai” pestle on the eight formations of “Baihui” for three minutes (every formation for 14 times), (3) apply down-up technique with the tip of a “Kuixing” pestle on the two side of “Fengchi” point for 7 times each. Then apply circulation technique with the handle of the “Kuixing” pestle on the two side of “Fengchi” point for 2 minutes each, (4) repeat step 3 on two sides of temple point, two sides of “Fengchi” point and “Yintang” point, (5) apply tapping technique with a "Wu Xing San Tai” pestle on the eight formations of “Shinto” (every formation for 7 times) and apply Tai-chi circulation technique with either a “Vajra” pestle or a “Qiyao Hunyuan” pestle for 14 times. Then apply opposite pushing technique with the tip of a “Qiyao Hunyuan” pestle for seven times. (Whole step for 5 minutes), (6) repeat step 5 on two sides of “Neiguan” point and two sides of “Fengchi” point, and (7) repeat step 5 on two sides of “Shenmen” point and two sides of “Fengchi” point.  **Dose:** 30 minutes, on the day of admission, the day before surgery, and 1 hour before the surgery. | Usual care | Anxiety (State-Trait Anxiety Inventory-state scale):   - Significantly lower in the IG than in the CG within preoperative 24 hours (Hedges' g = -1.29, *p* = 0.000).   Postoperative pain (Visual analogue Scale):   - Significantly lower in the IG than in the CG within postoperative 24 hours (Hedges' g = -0.93, *p* = 0.000).   Analgesic consumption:   - Significantly consumed less tramadol in the IG than in the CG at 24 hours after surgery (*p* = 0.039).   Bowel movement:   - Significantly shorter time in the IG than in the CG after surgery (Hedges' g = -0.68, *p* = 0.001).   Length of stay:   - Significantly shorter time in the IG than in the CG after surgery (Hedges' g = -0.29, *p* = 0.073). |
| Liao & Zhu.  (2022).  China | Sensory Stimulations (Tactile) | Elective laparoscopic cholecystectomy | 36:36 | **Content:** Usual care and intervention: Acupuncture by inserting the needle in sequence slowly and vertically in the order of Shenmen, Neiguan, Sanyinjiao and Taichong by the lifting, inserting and twisting technique.  **Dose:** 30 minutes, a total of three times of acupuncture performed preoperatively since admission | Usual care | Anxiety (State-Trait Anxiety Inventory-state scale):   - No significant difference between groups within preoperative 24 hours.   Analgesic Consumption:   - Significantly lower unspecified analgesics consumption in the IG than in the CG (*p* = 0.017).   Length of stay:   - No significant difference between groups. |
| Pasyar et al.  (2020).  Iran. | Sensory Stimulations (Olfactory) | Elective laparoscopic cholecystectomy | 30:30 | **Content:** Usual care and intervention: aromatherapy, 2 drops of bergamot orange essence (3%) were poured on a cotton ball attached to the patient’s collar for breathing.  **Dose:** 20 minutes, in the morning before surgery, once only. | Usual care and 2 drops of odorless grape seed oil were poured on a cotton ball attached to the patient’s collar for 20 minutes breathing. | Anxiety (State-Trait Anxiety Inventory-state scale):   - No significant difference between groups before surgery within preoperative 24 hours. |
| Yadegari et al.  (2022).  Iran | Sensory Stimulations (Olfactory) | Elective laparotomy | 42:42 | **Content:** Usual care and intervention: aromatherapy, 2 drops of jasmine essential oil (JEO) (50%) were poured on the patient’s collar. Patients were asked to inhale.  **Dose:** 60 minutes, in the morning before surgery, once only. | Usual care and 2 drops of sterile distilled water were poured on the patient’s collar for 60 minutes inhalation. | Anxiety (State-Trait Anxiety Inventory-state scale):   - Significantly lower in the IG than in the CG within preoperative 24 hours (Cohen's d = -1.82, *p* = 0.0000). |
| Abbasnia et al.  (2023).  Iran | Sensory Stimulations (Visual) | Elective laparoscopic cholecystectomy | 49:49:47 | **Content:** Usual care and intervention: Distraction images with self-selected 360^o^ video (nature, ocean, space) via virtual reality technology (Group 1) or simulation film of a preoperative scenario via virtual reality technology (Group 2).  **Dose:** 5 minutes, once only, before the surgery | Usual care. | Anxiety (State-Trait Anxiety Inventory-state scale):   - Significantly lower in the group 1 than in the CG within preoperative 24 hours (Hedges' g = -1.25, *p* < 0.001). - Significantly lower in the group 2 than in the CG within preoperative 24 hours (Hedges' g = -1.44, *p* < 0.001).   Postoperative pain (Visual analogue Scale):   - Significantly lower in the group 1 than in the CG within postoperative 24 hours (Hedges' g = -0.30, *p* <0.001). - Significantly lower in the group 2 than in the CG within postoperative 24 hours (Hedges' g = -0.58, *p* < 0.001). |
| Ugras et al.  (2023).  Turkey | Sensory Stimulations (Visual) | Elective colorectal and abdominal wall surgery | 43:43 | **Content:** Usual care and intervention: Delivering virtual reality (VR) via a VR headset and headphones with mobile phones. Then watched a playlist of five three-dimensional videos (E.g., a walk in the forest and park, beach trips, and space travel) with relaxing music in the background.  **Dose:** 10 minutes once only during the transfer from the surgery clinic to the operating room and in the preoperative holding area. | Usual care | Anxiety (Anxiety Specific to Surgery Questionnaire):   - Significantly lower in the IG than in the CG within preoperative 24 hours (Cohen's d = -0.72, *p* < 0.001). |
| Lin & Wang.  (2005).  Taiwan | Preoperative Counseling | Elective abdominal surgery | 32:30 | **Content**: Usual care and intervention: Preoperative counseling on postoperative pain includes education about the causes of pain and other uncomfortable feelings, the influence of pain, the importance of pain management and early out-of-bed activities, non-medical pain relief methods, the use of analgesics, and setting pain control goals. It allowed raising concerns and feelings and answering questions.  **Dose:** 20-30 mins, 1-3 days before surgery, once only | Usual care | Anxiety (Visual analogue Scale):   - Significantly lower in the IG than in the CG on one to three days before surgery (Hedges' g = -0.65, *p* < 0.001).   Postoperative pain (Visual analogue Scale):   - Significantly lower in the IG than in the CG within postoperative 24 hours (Hedges' g = -0.80, *p* < 0.05). |
| Sadati et al.  (2013).  Iran. | Preoperative Counseling | Elective laparoscopic cholecystectomy | 50:50 | **Content:** Preoperative counseling, including an introduction to the operating room environment, the surgical team, the anesthesia process, the benefits of laparoscopic versus open surgery, and postoperative care from the recovery room to patient discharge. It allowed raising concerns and feelings and answering questions.  **Dose:** On the day before surgery, once only. | Usual care | Anxiety (State-Trait Anxiety Inventory-state scale):   - Significantly lower in the IG than in the CG within preoperative 24 hours (*p* < 0.05).   Postoperative pain (Visual analogue Scale):   - Significantly lower in the IG than in the CG within postoperative 24 hours (*p* = 0.001).   Time to bowel movement:   - No significant difference between groups.   Length of stays:   - No significant difference between groups. |
| Lu & Zhang.  (2018).  China. | Preoperative Counseling | Elective hepatectomy | 45:45 | **Content:** Usual care and intervention: Preoperative counseling including, (1) encouraging to express their feelings of pain, anxiety, and prognosis during surgical treatment, (2) guiding them to identify one to two current issues and set goals, (3) providing the information regarding disease, treatment, and future plans while fostering positive thinking, (4) affirming family support and care, and (5) helping patients recognize the progress they’ve made and summarizing their experiences with appreciation.  **Dose:** Five sessions, one session per day, before surgery | Usual care | Anxiety (Self-Rating Anxiety Scale):   - Significantly lower in the IG than in the CG within preoperative 24 hours (Cohen's d = -1.05, *p* = 0.002). |
| Amini et al.  (2019).  Iran. | Information Provision | Elective hernia & cholecystectomy | 20:20:20 | **Content:** Usual care and intervention: Face-to-face verbal education (Group 1) or Self-read education booklet printed in white and black (Group 2), with the same content between the two groups, including introduction about surgery and elective surgeries, pathophysiology of hernia and biliary diseases, presurgical tests and their rational, routine for the night before the surgery and their logic, routine on the day of surgery with their logical reasons, and information related to Operating room, anesthesia process.  **Dose**: Group 1: Once only, on the day of admission. | Usual care | Anxiety (State-Trait Anxiety Inventory-state scale):   - Significantly lower in the group 1 than in the CG within preoperative 24 hours (Cohen's d = -0.84, *p* = 0.01). - Significantly lower in the group 2 than in the CG within preoperative 24 hours (Cohen's d = -0.70, *p* = 0.01). |
| Gade et al.  (2014). Norway | Psychological Intervention (Cognitive Behavioral Intervention) | Elective bariatric surgery | 48:50 | **Content:** Usual care and intervention: Eleven sessions of cognitive behavioral intervention with homework tasks in between the sessions. (First five sessions: face to face; the remaining six: telephone calls), including (Sessions 1-2) Strategies to enhance intrinsic motivation and address resistance to change, and (Sessions 2–11) Cognitive behavioral intervention  **Dose:** 10 weeks | Usual care. | Anxiety (Hospital Anxiety and Depression Scale – Anxiety scale):   - Significantly lower in the IG than in the CG one to two months before surgery (Hedges' g = -0.62, *p* = 0.003). |
| Cassina et al.  (2016).  Canada. | Psychological Intervention (Cognitive Behavioral Intervention) | Elective bariatric surgery | 23:24 | **Content:** Intervention: A total of six telephone-based cognitive behavioral intervention sessions were conducted with homework between each session. It focused on introduction of the cognitive behavioral model of overeating and obesity, scheduling meals at regular time intervals with recording consumption on food records, scheduling pleasurable alternative activities to overeating, identifying and planning for difficult eating scenarios, reducing vulnerability to overeating by solving problems and challenging negative thoughts, and preparation for the surgery.  **Dose**: 55 minutes, once per week, for six weeks | Usual care. | Anxiety (Generalized Anxiety Disorder-7):   - Significantly lower in the IG than in the CG one to two months before surgery (Hedges' g = -0.87, *p* < 0.001). |
| Felix et al.  (2018).  Brazil. | Psychological Intervention (Relaxation Exercise) | Elective laparoscopic bariatric surgery | 12:12 | **Content:** Usual care and intervention: guided imagery therapy conducted using a headset connected to a MP3 player, including the elements of soft background natural sound, guiding voice-over inviting participants to rest in a comfortable position and perform movements of slow and expansive breathing and relaxation in various parts of the body.  **Dose:** 20 minutes, once only, 24 hours before surgery. | Usual care and resting in the bed and using earphones without audio, connected to an MP3 player for 20 mins with the same dose. | Anxiety (State-Trait Anxiety Inventory-state scale):   - Significantly lower in the IG than in the CG within preoperative 24 hours (Cohen's d = -1.04, *p* = 0.005). |
| Menevşe & Yayla.  (2024).  Turkey. | Psychological Intervention (Relaxation Exercise) | Elective laparoscopic cholecystectomy | 56:56 | **Content:** Delivering energy therapy using the Emotional Freedom Technique (EFT), which includes providing information about EFT, and the 14 meridian points. Then performed it in three rounds without breaks in a quiet and calm environment, and in a comfortable position. The steps include asking about surgical fears and anxiety, formulating and speaking aloud a statement to cope with these feelings while tapping on the 14 meridian points from top to bottom. Each point is tapped ten times while repeating the statement and imagining and visualizing energy in their fingertips as it passes through their body. After that assume the heart-healing posture while focusing on their anxiety and fear, taking three deep breaths.  **Dose:** 25-30 minutes, once only, on the morning of surgery | Usual care | Anxiety (Anxiety Specific to Surgery Questionnaire):   - Significantly lower in the IG than in the CG within preoperative 24 hours (Cohen' d = -1.58, *p* = 0.000). |
| Garcia et al.  (2018).  Brazil. | Psychological Intervention (Preoperative Discussion) | Elective colorectal cancer surgery | 25:25 | **Content:** Intervention: Discussion with the researcher about their experience with hospitalization for cancer treatment (concerns, fears, doubts, or any other issue the patient wanted to treat).  **Dose:** 30 minutes, once only, on the day before surgery. | 30 minutes’ blank time with a conclusion of the research conducted by the researcher. | Anxiety (State-Trait Anxiety Inventory-state scale):   - No significant difference between groups within preoperative 24 hours. |
| Barberan-Garcia et al.  (2018).  Spain. | Prehabilitation | Elective major abdominal surgery | 63:62 | **Content:** Usual preoperative care includes a prehabilitation program that features a motivational interview and a personalized regimen. This regimen consists of sit-to-stand exercises, stair climbing, elastic band workouts, and indoor walking, with daily increasing steps and recording. Additionally, it includes a supervised high-intensity endurance exercise training program performed on a cycle ergometer, tailored to the individual.  **Dose**: 1 to 3 sessions per week, total for at least 4 weeks before surgery | Usual preoperative care | Anxiety (Hospital Anxiety and Depression Scale – Anxiety scale):   - No significant difference between groups before surgery (post-intervention).   Length of stay:   - No significant difference between groups. |
| ***Anxiety measuring time: After surgery*** | | | | | | |
| Tsay et al.  (2008).  Taiwan. | Sensory Stimulation (Tactile) | Elective gastric and liver cancer surgery | 30:31 | **Content:** Usual care with intervention: Delivering foot reflexotherapy by a reflexotherapist used the thumb and forefingers to apply pressures and massage to each reflex zone twice initially. Then apply and massage with techniques of kneading, friction, rubbing, and petrissage for 5 times or more at the digestive reflex zones of upper and lower abdomen, liver, spleen, gallbladder, duodenal, intestine, and colon.  **Dose**: 20 minutes, starting from the evening on postoperative day 2 (for 5 days) while patients are on PCA and between 1 and 3 hours after a dose of pain medication. | Usual care | Anxiety (Hospital Anxiety and Depression Scale – Anxiety scale):   - Significantly lower in the IG than in the CG on postoperative day 5 (Hedges' g = -0.77, *p* < 0.004).   Postoperative pain (Visual analogue Scale):   - Significantly lower in the IG than in the CG on postoperative day 3 (Hedges' g = -0.85, *p* < 0.001).   Analgesic consumption:   - Significantly lower use of narcotics in the IG than in the CG. (Hedges' g = -0.64, *p* = 0.015) |
| Demir & Saritas.  (2020).  Turkey. | Sensory Stimulation (Tactile) | Elective liver transplantation | 40:40 | **Content:** Usual care and intervention: delivering one-hand massage, with the following techniques: Petrissage, Friction, and Kneading.  **Dose:** 10 minutes, once only, after surgery | Usual care | Anxiety (State-Trait Anxiety Inventory-state scale):   - Significantly lower in the IG than in the CG after the surgery in the intensive care unit (Cohen's d = -1.35, *p* < 0.001).   Postoperative pain (Visual analogue Scale):   - Significantly lower in the IG than in the CG after the surgery in the intensive care unit (Cohen's d = -1.76, *p* < 0.001). |
| Soylu & Kartın.  (2021).  Turkey. | Sensory Stimulation (Tactile) | Elective laparoscopic cholecystectomy | 26:27 | **Content:** Intervention: Acupressure by applying pressure with the tips of the thumbs of both hands of the researchers for 3 minutes at the ST25, CV12, TH6, and HT7 acupuncture points (for 10 s continuously then rested for 2 s, and repeated this cycle for 3 minutes).  **Dose:** A total 12 minutes, at postoperative 0, 4, and 8 hours | A light touch was applied by the researchers to the same acupuncture points at the same time points for 1 s. | Anxiety (State-Trait Anxiety Inventory-state scale):   - No significant difference between groups within postoperative 24 hours.   Postoperative pain (Numeric rating scale):   - Significantly lower in the IG than the CG within postoperative 24 hours (*p* = 0.042).   Time to first bowel movement:   - No significant difference in time to first flatus between groups. |
| Nilsson et al.  (2005).  Sweden. | Sensory Stimulation (Auditory) | Elective hernia repair | 25:25:25 | **Content:** Usual care and intervention: Intraoperative (Group 1: Exposed to music intraoperatively and sham compact CD player postoperatively) VS Postoperative (Group 2: Exposed to sham compact CD player intraoperatively and music postoperatively), with the music, new-age synthesizer which was soft, relaxing, included seven different melodies, and with a total of 43 mins playing time.  **Dose:** Group 1: once only, auto-reverse mode from the end of anesthesia induction to the end of surgery with wound dressing; Group 2: auto-reverse mode from the time of arrival inward and for 1 hour. | Usual care and exposure to a sham CD player both intraoperatively and postoperatively. | Anxiety (Numeric rating scale):   - Significantly lower in group 1 than in the CG within postoperative 24 hours (Cohen's d = -0.51, *p* < 0.05). - Significantly lower in group 2 than in the CG within postoperative 24 hours (Cohen's d = -0.60, *p* < 0.05).   Postoperative pain (Numeric rating scale):   - Significantly lower in group 1 than in the CG within postoperative 24 hours. (Cohen's d = -0.75, *p* < 0.05). - Significantly lower in group 2 than in the CG within postoperative 24 hours. (Cohen's d = -0.99, *p* < 0.01).   Analgesic consumption:   - No significant difference of morphine consumptions between group 1 and the CG after 1 h in the PACU. - Significantly lower morphine consumptions in group 2 than in the control groups after 1 h in the PACU. (Cohen's d = -0.74, *p* < 0.05). |
| Akelma et al.  (2020).  Turkey. | Sensory Stimulation (Auditory) | Elective inguinal hernia surgery | 58:59 | **Content:** Usual care and intervention: Delivering preoperative music intervention via listening to their favorite music (50–60 dB) with headphones that completely covered their ears.  **Dose:** 15 minutes, once only, before the surgery. | Usual care | Anxiety (State-Trait Anxiety Inventory-state scale):   - Significantly lower in the IG than in the CG within postoperative 24 hours (Hedges' g = -0.54, *p* = 0.025).   Postoperative pain (Numeric rating scale):   - No significant difference within postoperative 24 hours. |
| Yu & Wang.  (2018).  China | Sensory Stimulation (Gustatory) | Elective hepatectomy |  | **Content:** Usual care and intervention: chewing a certain brand of sugar-free and cool mint flavored gum and rinsing mouth with warm water at two time points every day at 8:00am and 14:00pm.  **Dose**: 2 capsules/time, 15min/time (chew about 200 times), since postoperative day 1 until day 5 | Usual care | Anxiety (State-Trait Anxiety Inventory-full scale):   - Significantly lower in the IG than in the CG on postoperative day 5 (Cohen’s d = -1.83, *p* = 0.000).   Postoperative pan (EORTC QLQ-HCC18-pain scale):   - No significant difference between groups on postoperative day 5.   Length of stay:   - Significantly shorter in the IG than in the CG (Cohen’s d = -0.33, *p* = 0.014). |
| Lim et al.  (2019).  Singapore. | Preoperative Counseling | Elective colorectal surgery requiring stoma formation | 27:24 | **Content:** Usual care and intervention: Stoma psychosocial intervention: a pre‐operative individual face‐to‐face counseling session, an educational booklet provided, and five telephone follow‐ups (one pre‐operatively and four postoperatively) by a colorectal nurse.  **Dose:** Duration not available | Usual care. | Anxiety (Hospital Anxiety and Depression Scale – Anxiety scale):   - No significant difference between groups on postoperative day 3.   Length of stay:   - No significant difference between groups. |
| Goktas et al.  (2022).  Turkey. | Preoperative Counseling | Elective laparoscopic cholecystectomy | 30:30 | **Content:** Usual care and intervention: Delivering structured education by a multidisciplinary team consisting of a surgeon, an anesthesiologist, and a nurse. The content included preoperative preparation, anesthesia, intubation, mobilization, deep breathing, and coughing exercises, nutrition and fluid management, the postoperative recovery process, and clinical and operating room protocols. Lastly, it allowed raising concerns and feelings and answering questions. Written documents were provided that included the same topics.  **Dose:** 30 minutes, at least 12 hours before the surgery, once only. | Usual care. | Anxiety (State-Trait Anxiety Inventory-state scale):   - Significantly lower in the IG than in the CG within postoperative 24 hours (Cohen's d = -1.39, *p* < 0.001).   Postoperative pain (Visual analogue Scale):   - Significantly lower in the IG than in the CG within postoperative 24 hours (Cohen's d = -0.90, *p* = 0.01). |
| Klaiber et al.  (2018).  Germany. | Information Provision | Elective major visceral surgery | 138:106 | **Content:** Usual care with preoperative education seminar given by trained nursing staff, and a 48-page information brochure was provided, which included the same topics. Contents included (1) measures to prevent postoperative complications, (2) instructions about the principles of acute pain therapy and various coping strategies, (3) introduction of breathing exercises, postoperative out-of-bed mobilization, and practical exercises to prevent thrombosis and burst abdomen, and (4) explanation of the risks and preventive measures against in-hospital falls.  **Dose:** 1 hour, on the day before surgery, once only | Usual care and 48-page information brochure, which is the same with the interventional group. | Anxiety (Hospital Anxiety and Depression Scale – Anxiety scale):   - No significant difference between groups on postoperative day 7.   Postoperative pain (Brief Pain Inventory):   - No significant difference between groups on postoperative day 2.   Length of stay:   - No significant difference between groups. |
| Hasanpour-Dehkordi et al.  (2019).  Iran | Psychological Intervention (Relaxation Exercise) | Elective upper and lower gastrointestinal system surgery | 35:35 | **Content:** Usual care with the intervention: Benson’s relaxation technique, including (1) repeating a word (chosen by patients) always reminded him/her of calmness during relaxation, (2) performing with a specialized researcher during a 30 to 45-minute, face-to-face session with clarification of any ambiguities, and (3) afterward, performing with a guiding audio recording played via a MP3 player with headphones.  **Dose:** 20 minutes, every 6 hours for 2 days until 2 hours before the operation. | Usual care | Anxiety (State-Trait Anxiety Inventory-state scale):   - Significantly lower in the IG than in the CG within postoperative 24 hours (Cohen’s d = -0.97, *p* < 0.05).   Postoperative pain (Numeric rating scale):   - Significantly lower in the IG than in the CG within postoperative 24 hours (*p* < 0.003). |
| Lu et al.  (2022).  Taiwan. | Psychological Intervention (Relaxation Exercise) | Elective laparoscopic cholecystectomy | 33:33 | **Content:** Usual care and intervention: Delivering guided Imagery with a guiding CD with slow music in the background consisted of four important elements: (1) meditation practice; (2) guided images; (3) music assistance; and (4) 5-minute breathing relaxation training.  **Dose:** 15-20 minutes per session, once before surgery, and then, twice a day (in the morning and evening) after surgery, until the date of discharge. | Usual care | Anxiety (Beck Anxiety Inventory):   - Significantly lower in the IG than in the CG after surgery on postoperative day 2 (Cohen’s d = -0.81, *p* = 0.01).   Postoperative pain (Numeric rating scale):   - Significantly lower in the IG than in the CG on postoperative day 2 (Cohen’s d = -1.18, *p* < 0.001).   Analgesic consumption:   - No significant difference opioid or paracetamol consumptions between groups. |
| Ozhanli & Akyuz.  (2022).  Turkey. | Psychological Intervention (Relaxation Exercise) | Elective laparoscopic colorectal surgery | 31:32 | **Content:** Usual care and intervention: Progressive relaxation exercise, applied with the researchers, accompanied by video and music. Before the exercise, the definition, importance, purpose, and benefits of relaxation, explaining what to pay attention to, and teaching correct breathing techniques would be explained to patients.  **Dose:** 15 minutes, once preoperatively, and postoperative days 1, 2, and 3 after breathing exercise training. | Usual care | Anxiety (State-Trait Anxiety Inventory-state scale):   - Significantly lower in the IG than in the CG after surgery on postoperative day 3 (Hedges' g = -1.13, *p* = 0.00).   Postoperative pain (McGill Pain Questionnaire):   - Significantly lower in the IG than in the CG on postoperative day 3 (Hedges' g = -1.56, *p* = 0.00).   Analgesic consumption:   - Significantly lower Opioid consumptions in the IG than in the CG (Hedges' g = -0.81, *p* = 0.002). - No significant difference in non-steroid anti-inflammatory drug consumptions between groups. |
| Aktaş & İlgin.  (2023).  Turkey. | Psychological Intervention (Relaxation Exercise) | Elective bariatric surgery | 30:30:30 | **Content:** Usual care and intervention: 4-7-8 breathing technique (Group 1) VS deep breathing technique (Group 2)  **Dose:** Group 1: Applied between postoperative hours 1 and 6 as 1 set (4 breaths) per hour.; Group 2: Applied between postoperative hours 1 and 6 as 10 breaths per hour. | Usual care | Anxiety (State-Trait Anxiety Inventory-state scale):   - Significantly lower in the group 1 than in the CG within postoperative 24 hours (Cohen’s d = -1.52, *p* = 0.000). - Significantly lower in the group 2 than in the CG within postoperative 24 hours (Cohen’s d = -0.58, *p* = 0.01). |
| ***Anxiety measuring time: Before and After Surgery*** | | | | | | |
| Toğaç & Yılmaz.  (2021).  Turkey. | Preoperative Counseling | Elective laparoscopic cholecystectomy | 62:62 | **Content:** Usual care and intervention: individualized education program with audiovisual materials including a video, a leaflet, and an education booklet. The video included four stages: (1) Providing information about the disease, preoperative preparation, activity, surgery, complications, wound care, nutrition, and medicines, (2) Knowledge about the perioperative process, anesthesia, and surgical team was provided, (3) Photographs and leaflets about postoperative care were shown, and (4) Answer the questions raised by the patients with reinforcement.  **Dose:** 30-45 mins, once, on the day before surgery. | Usual care | Anxiety (State-Trait Anxiety Inventory-state scale):   - Significantly lower in the IG than in the CG within preoperative 24 hours (Cohen's d = -1.67, *p* = 0.000). - Significantly lower in the IG than in the CG within postoperative 24 hours (Cohen's d = -1.27, *p* = 0.000).   Postoperative pain (Visual analogue Scale):   - Significantly lower in the IG than in the CG within postoperative 24 hours (Cohen's d = -0.648, *p* = 0.000). |
| Lim et al.  (2011).  Singapore. | Information Provision | Elective breast and abdominal surgery (with subgroup analysis) | 112:114  (For abdominal surgery, 56:64) | **Content**: Usual care and intervention: Question prompt list related to patients’ illness and the coming surgery. Patients were encouraged to ask questions in the prompt list to the doctors.  **Dose**: One day before surgery, during ward rounds, once only. | Usual care with usual information related to the admission procedure. | Anxiety (State-Trait Anxiety Inventory-state scale):   - No significant difference between groups within preoperative 24 hours. - No significant difference between groups on postoperative day one to day three. |
| Tou et al.  (2012).  Australia. | Information Provision | Elective bowel surgery | 16:15 | **Content**: Usual care and intervention: A two-dimensional (2D) animation video presenting the patient’s complete in-hospital journey with an information sheet provided, including information about the role of the pre-admission clinic, bowel preparation, stoma information, bowel surgery, postoperative recovery, catheters, and pain control, etc.  **Dose:** 13 minutes, once, before the day of admission. | Usual care. | Anxiety (State-Trait Anxiety Inventory-full scale):   - No significant difference between groups within preoperative 24 hours. - No significant difference between groups within postoperative 24 hours. |
| Tusek et al.  (1997).  The U.S. | Psychological Intervention (Relaxation Exercise) | Elective colorectal surgery | 65:65 | **Content:** Usual care and intervention: Listening to a guided imagery tape, including the elements of (1) Soft, soothing, musical background, (2) Audio brought the patients to a “special place” in their minds which was safe, secure, protected, supported, and relaxed to them, and (3) an imagery story encourages patients to confront and work through any negative feelings.  **Dose**: Preoperative: Daily, 20 minutes, for 3 consecutive days; Intraoperative: audio-reverse 20-min music-only (same as the guided imagery tape) tape during induction of anesthesia, during the operation, and in the recovery room; Postoperative: daily for the first six consecutive days. | Usual care | Anxiety (Visual analogue Scale):   - Significantly lower in the IG than in the CG within preoperative 24 hours (*p* < 0.001). - Significantly lower in the IG than in the CG within postoperative 24 hours (*p* < 0.001).   Postoperative pain (Visual analogue Scale):   - Significantly lower in the IG than the CG within postoperative 24 hours (*p* < 0.001).   Analgesic consumption:   - Significantly lower narcotic analgesics consumptions in the IG than in the CG (*p* < 0.001).   Bowel movement   - Significantly faster in the IG than in the CG (*p* = 0.003)   Length of stay:   - No significant difference between groups. |
| Bulut & Karabulut.  (2023).  Turkey. | Psychological Intervention (Relaxation Exercise) | Elective laparoscopic cholecystectomy | 58:57 | **Content:** Usual care and intervention: Performing 5 repetitions of 10 breathing exercises with confirmation of the technique by the researcher  **Dose:** every 3 hours a day, between 09:00 and 21:00, 1 day before the surgery, on the day of the surgery, and on the first day after surgery. | Usual care | Anxiety (State-Trait Anxiety Inventory-state scale):   - Significantly lower in the IG than in the CG within preoperative 24 hours (Hedges' g = -0.75; *p* = 0.000). - Significantly lower in the IG than in the CG within postoperative 24 hours (Hedges' g = -1.25; *p* = 0.000). |
| **Notes**: IG: Interventional Group; CG: Control Group | | | | | | |
